# Dysregulated splicing factor SF3B1 unveils a dual therapeutic vulnerability to target pancreatic cancer cells and cancer stem cells with an anti-splicing drug

**DOI:** 10.1186/s13046-021-02153-9

**Published:** 2021-12-02

**Authors:** Emilia Alors-Perez, Ricardo Blázquez-Encinas, Sonia Alcalá, Cristina Viyuela-García, Sergio Pedraza-Arevalo, Vicente Herrero-Aguayo, Juan M. Jiménez-Vacas, Andrea Mafficini, Marina E. Sánchez-Frías, María T. Cano, Fernando Abollo-Jiménez, Juan A. Marín-Sanz, Pablo Cabezas-Sainz, Rita T. Lawlor, Claudio Luchini, Laura Sánchez, Juan M. Sánchez-Hidalgo, Sebastián Ventura, Laura Martin-Hijano, Manuel D. Gahete, Aldo Scarpa, Álvaro Arjona-Sánchez, Alejandro Ibáñez-Costa, Bruno Sainz, Raúl M. Luque, Justo P. Castaño

**Affiliations:** 1 Maimonides Biomedical Research Institute of Cordoba (IMIBIC), Córdoba, Spain; 2 Department of Cell Biology, Physiology, and Immunology, University of Cordoba, Córdoba, Spain; 3 Reina Sofia University Hospital, Córdoba, Spain; 4 CIBER Fisiopatología de la Obesidad y Nutrición (CIBERobn), Avenida Menéndez Pidal s/n, Edificio IMIBIC, 14004 Córdoba, Spain; 5 Department of Biochemistry, Universidad Autónoma de Madrid (UAM) and Department of Cancer Biology, Instituto de Investigaciones Biomédicas Alberto Sols (IIBM), CSIC-UAM, Madrid, Spain; 6 Department of Cancer Biology, Chronic Diseases and Cancer Area 3-Instituto Ramón y Cajal de Investigación Sanitaria (IRYCIS), Madrid, Spain; 7 Surgery Service, Reina Sofia University Hospital, Córdoba, Spain; 8 ARC-Net Research Centre, University and Hospital Trust of Verona, Verona, Italy; 9 Pathology Service, Reina Sofia University Hospital, Córdoba, Spain; 10 Medical Oncology Service, Reina Sofia University Hospital, Córdoba, Spain; 11 Department of Computer Sciences, University of Cordoba, Córdoba, Spain; 12 Department of Zoology, Genetics and Physical Anthropology, University of Santiago de Compostela, Lugo, Spain; 13 Department of Diagnostics and Public Health, Section of Pathology, University and Hospital Trust of Verona, Verona, Italy; 14 Centro de Investigación Biomédica en Red, Área Cáncer, CIBERONC, ISCIII, Madrid, Spain

**Keywords:** Pancreatic cancer, Splicing-spliceosome, SF3B1, Pladienolide-B, cancer stem cells

## Abstract

**Background:**

Pancreatic ductal adenocarcinoma (PDAC) is a highly lethal cancer, requiring novel treatments to target both cancer cells and cancer stem cells (CSCs). Altered splicing is emerging as both a novel cancer hallmark and an attractive therapeutic target. The core splicing factor SF3B1 is heavily altered in cancer and can be inhibited by Pladienolide-B, but its actionability in PDAC is unknown. We explored the presence and role of SF3B1 in PDAC and interrogated its potential as an actionable target.

**Methods:**

SF3B1 was analyzed in PDAC tissues, an RNA-seq dataset, and publicly available databases, examining associations with splicing alterations and key features/genes. Functional assays in PDAC cell lines and PDX-derived CSCs served to test Pladienolide-B treatment effects in vitro, and in vivo in zebrafish and mice.

**Results:**

*SF3B1* was overexpressed in human PDAC and associated with tumor grade and lymph-node involvement. *SF3B1* levels closely associated with distinct splicing event profiles and expression of key PDAC players (*KRAS, TP53*). In PDAC cells, Pladienolide-B increased apoptosis and decreased multiple tumor-related features, including cell proliferation, migration, and colony/sphere formation, altering AKT and JNK signaling, and favoring proapoptotic splicing variants (*BCL-XS/BCL-XL, KRASa/KRAS, Δ133TP53/TP53*). Importantly, Pladienolide-B similarly impaired CSCs, reducing their stemness capacity and increasing their sensitivity to chemotherapy. Pladienolide-B also reduced PDAC/CSCs xenograft tumor growth in vivo in zebrafish and in mice.

**Conclusion:**

SF3B1 overexpression represents a therapeutic vulnerability in PDAC, as altered splicing can be targeted with Pladienolide-B both in cancer cells and CSCs, paving the way for novel therapies for this lethal cancer.

**Supplementary Information:**

The online version contains supplementary material available at 10.1186/s13046-021-02153-9.

## Background

Pancreatic ductal adenocarcinoma (PDAC) represents 95% of all pancreatic tumors and is one of the most lethal cancers [1]. Unlike most cancers, life expectancy has only marginally improved for PDAC patients in the last decades. Current statistics report 5-year survival rates of 9% [1]. This, coupled to its increasing incidence, predicts that PDAC will become the second overall leading cause of cancer-related death in western countries by 2030 [2]. This dismal prognosis is the combined result of late diagnosis, absence of specific symptoms, lack of (bio)markers for both screening and early detection, and poor response to available therapeutic strategies (i.e., surgery, chemotherapy, and radiotherapy). The latter is due to the complex biological (molecular, cellular, and histological) architecture of this tumor [3]. Thus, despite remarkable advances in understanding PDAC, we have yet to discover broad actionable vulnerabilities, like *BRCA1*/*BRCA2* mutations affecting only a small percentage of patients, but whom can benefit from PARP inhibitor therapy [1]. Hence, new treatments for PDAC are urgently needed.

International collaborative studies have provided a comprehensive picture of the genomic landscape of PDAC, revealing recurrent mutations in four genes, *KRAS, CDKN2A, TP53*, and *SMAD4*, which are still (in general) clinically undruggable [3–5]. These efforts have also unveiled a few genes mutated in lower but appreciable proportions of cases, some of which (like the aforementioned *BRCA2*) are targetable [3, 4]. Interestingly, some of these genes (*U2AF1*, *SF3B1*, *RBM10*) are core players of alternative splicing, a key cellular process whose alteration is emerging as a widespread hallmark in every cancer studied and, most importantly, an attractive therapeutic target [6–8]. Earlier work identified the dysregulation of certain spliceosome components and splice variants (e.g., *CD44, MUC4, CCKBR*) in PDAC [9]. Subsequently, transcriptomic and in silico analyses indicated that splicing-related dysregulation involves altered patterns of splicing variants with potential tumorigenic, prognostic, survival, and immune implications [10]. Furthermore, PDAC mutational features and splicing alterations may be tightly interrelated as indicated by a recent study that elegantly dissected the complex interplay by which mutant p53 enhances KRAS signaling by increasing the expression of the splicing factor *HNRNPK* [11]. These findings emphasize the importance of better understanding the underpinnings of altered splicing in PDAC.

The Splicing Factor 3B Subunit 1 (*SF3B1*) is a spliceosome component essential in pre-RNA processing and the most frequently mutated splicing factor across cancers, particularly in hematological malignancies but also in solid tumors, including PDAC (although at a much lower frequency, 4% of cases) [3, 4, 12] (reviewed in [7, 13, 14]). *SF3B1* encodes for a core component of the U2 small nuclear ribonuclear protein (snRNP) and is required for the splicing of most introns, being involved in the recognition of the branch-site, an early stage of spliceosome assembly [7, 9, 13, 14]. Somatic mutations in *SF3B1* in cancer alter the correct recognition of pre-RNA patterns by the spliceosome due to reduced fidelity of branch-point selection and has recently been found to promote tumor glycolysis in PDAC [7, 13–15]. However, the pathological importance of *SF3B1* does not only rely on the well characterized role of *SF3B1* mutations, but growing evidence indicates that alteration of its expression can also have malignant consequences in some cancers, such as prostate cancer [16] and hepatocarcinoma [17]. These studies also underscore the potential of altered SF3B1 as a therapeutic target, as several drugs like Pladienolide-B (a macrocyclic lactone produced by *Streptomyces* sp.) and its derivatives can inhibit SF3B1 function and thereby exert antitumoral effects in several cancers [16, 17].

To date, expression of *SF3B1* and its potential as a therapeutic target have not been explored in detail in PDAC nor in pancreatic cancer stem cells (CSCs), a small population of undifferentiated cells capable of initiating tumor generation, differentiation, and self-renewal, and thus, are key drivers of tumor evolution, metastasis, and relapse [18, 19]. CSCs comprise distinct subsets with inherent characteristics, such as autofluorescence activity or the expression of specific cell surface antigens and receptors (mainly CD133, EpCAM, CXCR4 or CD44) [20]. Currently, new approaches seek to increase the susceptibility of CSCs to conventional treatments by identifying novel vulnerabilities in these cells. To date, only little evidence suggests splicing dysregulation in PDAC CSCs [21]. Therefore, we aimed to investigate the presence and role of SF3B1 in PDAC as well as its potential value as a therapeutic target. Towards this end, we assessed *SF3B1* expression in human tumor samples and in RNA-seq datasets, examined the molecular associations of *SF3B1* with splicing and mutational features by biocomputational approaches, and tested the functional consequences of modulating SF3B1 with Pladienolide-B in both PDAC cell line models and cell lines enriched in CSCs established from PDAC patient-derived xenografts (PDX). Our results demonstrate a dysregulation of SF3B1 in PDAC and unveil this protein as an actionable target in both PDAC cells and CSCs.

## Methods

### Patient samples

The present study was performed using 150 formalin-fixed paraffin-embedded (FFPE) samples (75 PDAC tumor and 75 corresponding non-tumor adjacent tissue (NTAT) and 24 fresh PDAC samples, which were obtained from surgical resections. Clinicopathological data of the FFPE cohort are described in Table 1 and Supplemental Table 1. Histological and immunohistochemical studies were performed separately by two experienced pathologists to identify tumor and NTAT. The Ethics Committee of the Reina Sofia University Hospital (Córdoba, Spain) approved the study, which was conducted in accordance with the Declaration of Helsinki. Patient clinical parameters were collected. Written informed consent was signed by every patient. FFPE samples were obtained from the Andalusian Biobank. Gene expression data were downloaded from public Array Express database [E-MTAB-1791] [22], GSE15471 [19], “The Cancer Genome Atlas” (TCGA) using cBioPortal (PanCancer Atlas) [5], and GSE79670 [23].

**Table 1:** Clinical characteristics of patients included

Characteristic	Samples (***n*** = 75)
**Age**	
	Median 65, (range 32–76)
**Sex**	
Female	22 (29.3)
Male	53 (70.7)
**T stage**	
T1	5 (6.7)
T2	14 (18.79)
T3	43 (57.39)
T4	9 (12)
NA	4 (5.3)
**N stage**	
N0	25 (33.3)
N1	46 (61.3)
NA	4 (5.3)
**M stage**	
M0	62 (82.7)
M1	7 (9.3)
MX	2 (2.7)
NA	4 (5.3)

*NA* Not Available

### Gene expression and splicing variants analysis

RNA-seq data produced generated from fresh-frozen tumor tissue of an additional cohort of 94 PDAC samples were analyzed to explore determined *SF3B1* expression and splicing profiles. Briefly, all samples were fresh frozen, and RNA was isolated using the miRNeasy Mini Kit (Qiagen, Milan, Italy), sample quantification was performed using a Qubit and Bioanalyzer to confirm quantitation and quality, respectively. RNAseq libraries were generated using RiboZero rRNA depletion followed by RNA library prep using NEBNext Ultra RNA Directional kit. IIlumina HiSeq2500 v4 was used and libraries were sequenced using PE 75 cycles at 7 samples per lane (> 50 million reads per sample). Clinicopathological data of the cohort are described in Supplemental Table 2. Patient samples and data were collected from the ARC-Net Research Centre, University of Verona, Italy, under approval number CE2172 (Prot 26,773) from the Integrated University Hospital Trust Ethics Committee. This dataset, used and analyzed during the current study, is available from the corresponding author upon reasonable request. Raw paired-end FASTQ files were quantified using Salmon [24] and the last release (v34) of the human GENCODE transcriptome [25]. The relative abundance of transcripts in transcripts per million (TPM) generated by Salmon were used as input for SUPPA2 software [26] to perform the calculation of relative abundances of the splicing events as Percent Spliced In Index (PSI or Ψ). To perform a clustering for SF3B1 expression, the Salmon quant-files were imported to R [27] and summarized to gene-level using Tximeta [28]. The gene abundances were imported to EdgeR [29, 30] and normalized by the trimmed mean of M-values (TMM) method [31]. TMM-normalized expression values of SF3B1 were used to classify the patients according to their expression using mclust [32] into groups using mclust E model (univariate, equal variance), which generated three groups labelled as low, intermediate, and high expression. Subsequently, PSI and TPM values for the low and high SF3B1 expression groups were used with SUPPA2 to perform the differential splicing analysis with local events, then splicing differences using delta PSI (ΔΨ) were calculated. The difference in average PSI from each group with adjusted, and *p* value < 0.05 were considered significant.

The PSI values were used to calculate the relative frequency of each splicing event per sample [Relative Frecuency (event i) = (Σ PSI (event i))/(Σ PSI (total events))] and estimate the splicing event composition per sample. The comparison between the SF3B1 high and low groups was tested by Wilcoxon test and Kolmogorov-Smirnov test with significance cutoff at *p* < 0.05. Classification of SE profiling was established into 7 types of events according to their splicing pattern: skipped exon, mutually exclusive exons, alternative 5′ splice site, alternative 3′ splice site, retained intron, alternative first exon, and alternative last exon (as illustrated in Fig. 2B).

### Cell lines culture and reagents

For functional assays, we used the non-tumoral pancreas-derived HPDE E6E7 cell line [generously provided by Dr. F.X. Real, Spanish National Cancer Centre (CNIO), Madrid, Spain] as a control, and three acquired PDAC model cell lines, Capan-2, BxPC-3, and MIAPaCa-2 (ATCC, Barcelona, Spain). In brief, cells were checked for mycoplasma contamination by PCR as previously reported [33]. The HPDE E6E7 cell line was cultured in Keratinocyte Serum Free Medium (Gibco, Madrid, Spain) containing two mandatory additives [(bovine pituitary extract (BPE) and human recombinant epidermal growth factor (EGF)] and 1% antibiotic-antimycotic (Gentamicin/Amphotericin B; Life Technologies). Capan-2 cells were cultured in McCoy’s 5A Medium (Gibco) supplemented with 10% fetal bovine serum (FBS, Sigma-Aldrich, Madrid, Spain), 2 mM L-glutamine (Sigma-Aldrich) and 0.2% antibiotic-antimycotic. BxPC-3 cells were cultured in RPMI 1640 medium (Lonza, Basel, Switzerland) with 2 mM L-glutamine, and 0.2% antibiotic-antimycotic. MIAPaCa-2 cells were cultured in Dulbecco’s Modified Eagle’s Medium with 4,500 mg/L of glucose (DMEM 4.5 g/l glucose) supplemented with 10% FBS, 2.5% Horse Serum [34], 2 mM L-glutamine and 0.2% antibiotic-antimycotic. Cell lines grew in a constant humidified 37 °C atmosphere with 5.0% CO_2_. Pladienolide-B (Santa Cruz Biotechnology, Bergheimer, Germany) was resuspended in DMSO and was initially used in the 0.01–100 nM range. Gemcitabine (Santa Cruz Biotechnology) was used at a concentration of 100 nM.

### PDX-derived tumor cell lines and CSC-enriching cultures

PDAC patient-derived xenografts (PDAC PDX) were obtained from Dr. Manuel Hidalgo under a Material Transfer Agreement with the CNIO, Madrid, Spain (Reference no. I409181220BSMH) and were originally described and genetically characterized [20]. To establish primary A6L, 215, 253 and 354 PDX-derived cultures, PDXs were enzymatically digested, resuspended and cultured in RPMI 1640 medium supplemented with 10% FBS and 50 U/mL penicillin/streptomycin. All cultures were tested for mycoplasma at least every 4 weeks.

To enrich for CSCs, 1000 cells from each cell line were seeded in 24-well Corning Costar ultra-low attachment plates (Merck, Madrid, Spain) to avoid cell attachment and differentiation. Cells were cultured in DMEM-F12 (Thermo Fisher, Madrid, Spain) supplemented with B-27 (Gibco) and FGF (PreproTech EC, London, U.K.). Numbers of spheres were determined by microscopy using an inverted EVOS FL microscope (Thermo Fisher) with a 10X objective with phase contrast.

### Alteration of *SF3B1* expression by specific siRNA

HPDE E6E7, Capan-2, BxPC-3, and MIAPaCa-2 cells were transfected with an SF3B1 specific siRNA previously validated in our laboratory (s23851; Thermo Fisher) [16, 17]. Specifically, cells were seeded in 6-well culture plates and transfected with the SF3B1 siRNA (75 nM) using Lipofectamine RNAiMAX Transfection Reagent (Invitrogen, Thermo Fisher), following the manufacturer’s instructions. A scrambled siRNA was used as a control. Silencing efficiency was validated by quantitative-PCR (qPCR). The experiments were performed in triplicate per cell line on independent days.

### Proliferation assay

To evaluate cell proliferation in response of 1 nM Pladienolide-B, 3000 cells/well (*n* = 4 well/treatment) were grown and compared with vehicle-treated-controls using Alamar-Blue reagent (Thermo Fisher) as previously reported [35].

### Wound-healing assay

The ability of HPDE E6E7 and MIAPaCa-2 cell lines to migrate after Pladienolide-B treatment (24 h) was evaluated in a wound-healing assay as previously reported [35].

### Colony formation assay

Colony formation was evaluated on MIAPaCa-2 and PDX-derived cell lines in response to Pladienolide-B treatment. Cells were treated for 24, 48 or 72 h with vehicle or Pladienolide-B, thereafter, 5000 (MIAPaCa-2 cells) or 2000 (PDX-derived cell lines) cells were seeded in 6-well plates and incubated for 10-days, changing medium every 3-days. After incubation, cells were fixed with Crystal Violet. MIAPaCa-2 colony numbers were evaluated using ImageJ-1.51 s software. PDX-derived cell lines were washed and incubated with 500 μL 1X PBS containing 10% SDS. Colonies lysates were colometrically examined at 520 nm (Synergy™-HT-Multi-Mode Microplate-Reader; BioTek, Winooski, Vermont, USA).

### Apoptosis assay

To evaluate the apoptotic rate for PDAC cell lines, 5000 cells/well were seeded in white 96-well plates and cultured for 24 h with Pladienolide-B or vehicle, and apoptotic rates were measured using Caspase-Glo 3/7 Reagent (Promega), following the manufacturer’s instructions [36]. For Annexin-V staining, floating and attached cells were pooled and resuspended in 1X Annexin-V staining buffer containing Annexin-V-FITC diluted 1:20 (Cat no. 29001, Biotium, Freemont, CA) and then, incubated for 20 min at room temperature prior to flow cytometric analysis. Cytometry data was acquired with an Invitrogen™ Attune™ NxT 4-laser cytometer with software version 3.1.1.

### Flow cytometry

Primary pancreatic cells (monolayers and spheres) were trypsinized and resuspended in Sorting Buffer (3 μM EDTA, and 3% FBS in 1X PBS). To identify CD133 positive CSC, the following conjugated antibodies were used: anti-CD133/1-APC or PE; (Miltenyi), and appropriate isotype-matched control antibodies. For autofluorescent detection, cells were excited with blue laser 488 nm and selected as the intersection with the filters 530/30 (BL1) and 590/40 (BL2) [20]. For all assays, 2 mg/mL DAPI (Cat no. D9564, Sigma-Aldrich) was used to exclude dead cells with laser VL1. Data were analyzed with FlowJo 9.3 software (Tree Star Inc., Ashland, OR.). Cytometry data was acquired with an Invitrogen™ Attune™ NxT 4-laser cytometer with software version 3.1.1.

### Cytotoxicity assay

To evaluate drug cytotoxicity, the Toxilight BioAssay kit was used (Lonza, Walkersville, MD), a bioluminescence-based assay which measures adenylate kinase released from damaged cells into culture medium.

### RNA extraction and reverse transcription

Total RNA from FFPE samples was extracted using Maxwell MDx 16 Instrument (Promega, Madrid, Spain) with the Maxwell 16 LEV RNA FFPE Kit (Promega, Madison, USA) according to the manufacturer’s instructions. Total RNA was isolated from PDAC cell lines and PDX-derived PDAC cell lines using TRIzol Reagent (Invitrogen, Barcelona, Spain) following the manufacturer’s instructions, and was treated with DNase (Promega, Barcelona, Spain). In every case, the amount of RNA recovered and its purity (before and after DNase treatment) was determined using the NanoDrop2000 (Thermo Fisher). One μg of RNA was reverse transcribed to cDNA using random hexamer primers [First Strand Synthesis (MRI Fermentas, Hanover, MD)] in a 20 μL volume.

### qPCR

qPCR reactions were performed using the Brilliant III SYBR Green-QPCR MasterMix (Stratagene, La Jolla, CA) in the Stratagene Mx3000p system as previously described [36]. Specific primers for transcripts studied were designed with Primer3 and Primer Blast software (Supplemental Table 3). Gene expression values were normalized to beta-actin (*ACTB*) mRNA levels, where *ACTB* did not show significant differences across conditions (data not shown).

### qPCR dynamic array based on microfluidic technology

A quantitative PCR dynamic array based on microfluidic technology was used to simultaneously measure the expression of 48 genes in 48 samples of fresh PDAC tumor samples (*n* = 24) and PDAC cell lines, as previously reported by our group [37]. Biomark System and FluidigmVR Real-Time PCR Analysis Software v.3.0.2 and Data Collection Software v.3.1.2 (Fluidigm) were used to obtain RNA expression levels in these samples. Primers for specific human transcript variants were designed with Primer3 and Primer Blast software (see Supplemental Table 4). RNA expression levels were normalized using the β-actin housekeeping gene (*ACTB*).

### Immunohistochemistry (IHC) analysis

IHC analysis was performed on FFPE PDAC sections (*n* = 18), comprising tumor and NTAT, using ImmPRESS-UNIVERSAL REAGENT Anti-Mouse/Rabbit IgG PEROXIDASE (Vector Laboratories, Maravai LifeSciences, Barcelona, Spain), SF3B1 monoclonal antibody (1:250; ab172634, Abcam, Cambridge, UK). Staining was evaluated in nuclei by assessing a combined score comprising the percentage of positive cells (0% = 0, 1–25% = 1, 26–50% = 2, 51–75% = 3, 76–100% = 4) multiplied by the intensity (no = 0, weak = 1, moderate = 2, strong = 3), ranging from 0 to 12 [38].

### Confocal microscopy

SF3B1 was analyzed in HPDE E6E7 and MIAPaCa-2 cell lines after 24 h of treatment with vehicle or Pladienolide-B, and after SF3B1 silencing. Briefly, cell lines were grown in glass coverslips and fixed with 4% PFA. SF3B1 (1:250) (ab172634, Abcam) Wheat Germ Agglutinin, Alexa Fluor 647 Conjugate (W32466, Thermo Fisher) was used to label membrane cells (1:300), Alexa Fluor 488 Donkey anti-Rabbit secondary antibody (1:500) (A-21206, Thermo Fisher), and nuclei were stained with 4′,6-diamidino-2-phenylindole (DAPI) (Sigma-Aldrich). Samples were visualized with a LSM710 confocal laser-scanning microscope (Carl Zeiss, Jena, Germany; Microscopy facility, IMIBIC), images were processed using the Huygens Essential software package (version 2.4.4; SVI, Hilversum, The Netherlands), and analyzed with ImageJ to study SF3B1 cell distribution.

### Western blotting

Cells were cultured (250,000/well, 12-well plates) for 24 h with Pladienolide-B or vehicle. Then, medium was removed and 300 μL of pre-warmed SDS-DTT (at 65 °C) was added to lyse the cells. Samples were sonicated for 10 s and boiled for 5 min at 95 °C. Extracted protein samples were separated in 12.5% polyacrylamide gels by SDS-PAGE, transferred to a nitrocellulose membrane (Ref. 1,704,270, Millipore) and blocked with 5% non-fat dry milk in Tris-buffered saline with 0.05% Tween-20 (Ref. 93,773, Sigma-Aldrich). Membranes were then incubated with the following primary antibodies: phospho-ERK1/2 (Ref. 4370S, Cell Signaling Technology; Danvers, Massachusetts), phospho-AKT (Ref. 9271S, Cell Signaling Technology), phospho-JNK (Ref. AF1205, R&D Systems; Minneapolis, Minnesota), total ERK1/2 (SC-154, Santa Cruz Biotechnology; Santa Cruz, California), total AKT (Ref. 9272S, Cell Signaling Technology), total JNK (Ref. AF1387, R&D Systems). Then, horseradish peroxidase-conjugated goat anti-rabbit IgG (Ref. 7074, Cell Signaling Technology) was used. Bond antibodies were visualized using the Clarity Western-ECL Blotting Substrate (Bio-Rad Laboratories, Madrid, Spain) and scanned using ImageQuant Las 4000 system (GE Healthcare Europe GmbH). Images were analyzed using ImageJ-1.51 s software.

### Zebrafish breeding, in vivo xenograft assays and image analysis

Zebrafish embryos were obtained by crossing adults (*Danio rerio*, wild type). Zebrafish adults were maintained in 30 L aquaria with a ratio of 1 fish/liter of water, a 14:10 day/night cycle and a water temperature of ≈ 28.5 °C, according to published procedures [39]. All procedures used in the experiments, fish care and treatment were performed in agreement with the Animal Care and Use Committee of the University of Santiago de Compostela and the standard protocols of Spain (Directive 2012–63-DaUE). At the final point of the experiments, zebrafish embryos were euthanized by tricaine overdose.

Collection of the zebrafish embryos occurred at 0 hpf (hours post fertilization). After that, eggs were incubated at 28.5 °C until 48 hpf. At this point, hatched embryos were anesthetized with 0.003% of tricaine (Sigma-Aldrich) and injected with MIAPaCa-2 or A6L cells, stably infected with an mCherry-H2B expressing lentivirus as previously described [40], under different treatment conditions (control and Pladienolide-B treated; 1 nM). MIAPaCa-2-mCherry-H2B and A6L-mCherry-H2B cells were incubated at 37 °C and 5% CO_2_ before injection until they reached a confluence of 70%. MIAPaCa-2-mCherry-H2B and A6L-mCherry-H2B cell preparations consisted of cells trypsinized and concentrated in a vial at a rate of 10^6^ cells per tube for each condition and resuspended in 10 μL of PBS with 2% of polyvinylpyrrolidone (PVP) to avoid cellular aggregation. For cell injection, borosilicate needles (1 mm O.D. × 0.75 mm I.D.; World Precision Instruments) were used. Between 100 and 200 cells were injected into circulation in each embryo (Duct of Cuvier) using a microinjector (IM-31 Electric Microinjector, Narishige) with an output pressure of 15 kPA and 10 ms of injection time per injection. Afterwards, embryos were incubated for 6 days post injection (dpi) at 34 °C in 30 mL Petri dishes with SDTW (Salt Dechlorinate Tap Water). Imaging of the injected embryos were performed using a fluorescence stereomicroscope (AZ-100, Nikon) at 1, 4 and 6 dpi to measure the spreading and proliferation of the injected cells in circulation in the zebrafish for each of the conditions assayed. Quantifish software [41] was used to perform the image analysis of the photographs taken of the embryos at 1, 4 and 6 dpi. Quantifish measures, in each of the images provided, the intensity of the fluorescence and the area of the positive pixel above a certain threshold of the cells. With these parameters, integrated density is obtained allowing for the comparison of different times between images to obtain a proliferation ratio of the cells in the region of the caudal hematopoietic tissue (CHT) of the embryos, where the cells metastasize.

### Xenograft mice model

Two × 10^6^ MIAPaCa-2 cells, resuspended in 100 μL of basement membrane extract, were injected in each flank of 7-week-old male athymic BALB/cAnNRj-Foxn1nu mice (Janvier Labs, Le Genest-Saint-Isle, France; *n* = 5 mice). Tumor growth was monitored twice/week for 7-weeks. At the fourth week of grafting, mice were injected intratumorally with 100 μL of Pladienolide-B. After euthanasia of mice, each tumor was dissected, fixed, and sectioned for histopathologic examination of necrosis after H&E staining by expert pathologists. A piece from each tumor was frozen for RNA extraction. These experiments were performed according to the European-Regulations for Animal-Care under the approval of the University of Cordoba research ethics committees (No. 15-05-2018-088).

### Statistical analysis

Samples from all groups were processed at the same time. Statistical differences between two variables were calculated according to normality, assessed by Kolmogorov-Smirnov test, using parametric t-test or non-parametric Mann Whitney U test. For groups with three or more variables, One-Way ANOVA analysis or Kruskal-Wallis test were performed. To normalize values within treatment and control groups and minimize intragroup variations in the different experiments, the values obtained were compared with controls (set at 100%). Results from in vitro studies were obtained from at least 3 separate independent experiments carried out on different days with different cell preparations. Data were expressed as mean ± SEM, *p* < 0.05 was considered statistically significant. Analyses were performed with SPSS v.22 (IBM SPSS Statistics Inc., Chicago, IL, USA) and GraphPad Prism 7 (GraphPad Software, La Jolla, CA, USA).

## Results

### Expression of *SF3B1* in PDAC

**Fig. 1:**
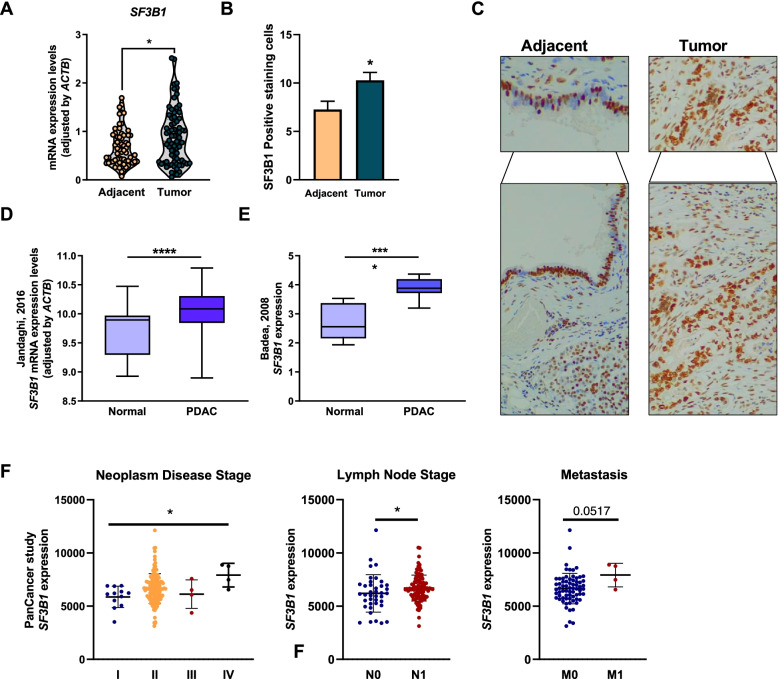
*SF3B1* expression in PDAC. **A** mRNA levels of *SF3B1* adjusted for *ACTB* gene expression in PDAC FFPE samples compared with non-tumoral adjacent tissue (NTAT). **B** SF3B1 IHC analysis in PDAC FFPE samples vs. NTAT. **C** Representative IHC 20X-image; SF3B1 nuclear immunostaining in non-tumoral adjacent tissue is evident in acinar and ductal cells (left panel) and in cancer cells (right panel). **D** *SF3B1* mRNA levels in E-MTAB-1791 [22] comparing PDAC and healthy controls. **E** *SF3B1* mRNA levels in GSE15471 [19] comparing PDAC and NTAT used as a control. **F** Correlation of *SF3B1* mRNA levels with clinical stage, lymph node involvement and distant metastasis (according to WHO) in PanCancer cohort [5]. Data represents mean ± SEM. Asterisks indicate significant differences (*p < 0.05; **p < 0.01; ***p < 0.001)

Expression levels of *SF3B1* were evaluated by qPCR in RNA isolated from FFPE samples from a cohort of 75 PDAC patients. Main clinical parameters are shown in Table 1 (see additional data in Supplemental Table 1). For each patient, tumor tissue was compared with its corresponding NTAT, used as reference. Results revealed that *SF3B1* mRNA expression levels were higher in PDAC tumor tissue compared with NTAT (Fig. 1A). Accordingly, IHC staining of 18 randomly selected samples from this same cohort revealed SF3B1 nuclear immunostaining in NTAT (acinar and ductal cells) and cancer cells, where the staining score was higher (Fig. 1B-C). Low *SF3B1* expression levels were associated with arterial hypertension (AHT) and type 2 diabetes mellitus (T2DM) in this patient cohort (Supplemental Fig. 1).

To validate our results, *SF3B1* mRNA levels were analyzed in publicly accessible datasets from human samples, the E-MTAB-1791 database (195 PDAC patients and 41 healthy controls) [22], and GSE15471 (36 PDAC samples and corresponding NTAT) [19]. In line with our results, *SF3B1* was overexpressed in both cohorts (Fig. 1D: E-MTAB-1791; Fig. 1E*:* GSE15471). Interestingly, accessible data from the PanCancer study (TCGA) [5] demonstrated that *SF3B1* expression levels were directly associated with neoplasm disease stage, being most expressed in poorly differentiated tumors (Fig. 1F). Moreover, *SF3B1* levels were directly associated with lymph node stage, tending to correlate with metastasis (despite the low number of metastatic patients available; Fig. 1F).

**Fig. 2:**
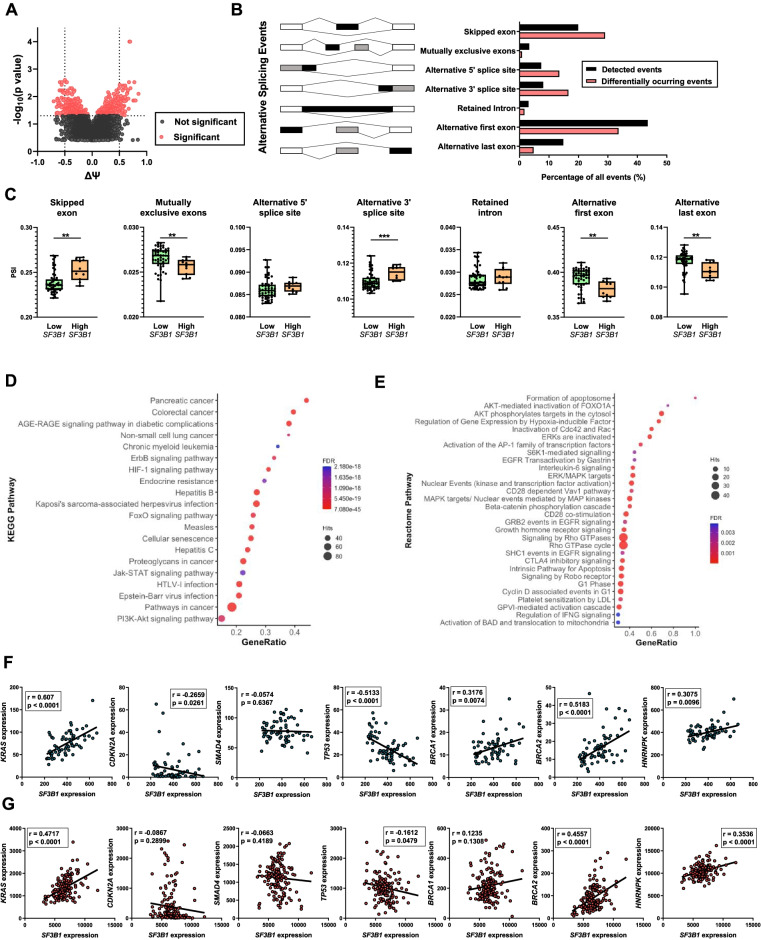
Relationship of *SF3B1* expression levels with splicing event patterns of key genes in PDAC. **A** Volcano-plot where ΔΨ of total events calculated is plotted against the –log10 *p*-value of the Fisher’s Exact Test to assay differential splicing events between high and low *SF3B1* expression groups of samples, showing that *SF3B1* tumor expression may influence alternative splicing pattern. **B** Alternative Splicing event characterization of RNA-seq samples. Total splicing events detected (black) and significantly different events between *SF3B1* expression groups (red) are classified depending on their type, showing different frequencies (%) between both conditions. **C** Significantly different Alternative Splicing Events comparison of PSI values between High and Low expression groups. **D** KEGG analysis of significantly different spliced genes depending on SF3B1 expression. Ratio of the genes’ hits over the total genes of a pathway (X-axis) is plotted for each pathway (Y-axis). The size of each point denotes the genes hits, and the color represents their significancy. **E** Reactome analysis of significantly different spliced genes depending on *SF3B1* expression. **F**, **G** Correlations between *SF3B1* and *KRAS, CDKN2A*, *SMAD4*, *TP53, BRCA1*, *BRCA2*, and *HNRNPK* mRNA levels in our RNA-seq cohort (**F**) and PanCancer cohort (**G**)

The potential impact of SF3B1 expression on alternative splicing in PDAC was assessed with a biocomputational approach that analyzed RNA-seq data of 94 additional PDAC patient samples (Supplemental Table 2), enabling the identification and quantification of splicing events. Samples were first classified into different clusters according to their SF3B1 expression levels, then the means of the Ψ of each event were compared between groups with high and low expression. This approach detected 482 splicing events that were significantly different according to *p* value and ΔΨ of the total of 240,941 events detected using SUPPA2 (Fig. 2A). Indeed, the general pattern of splicing events differed depending on SF3B1 expression levels, as these significantly different events displayed a higher frequency of skipped exons, alternative 3′ splice sites and alternative 5′ splice sites, and lower frequency of alternative first or last exons, compared to the average of all the events calculated (Fig. 2B). We used an additional software, rMATs, where we observed a similar pattern of splicing, specifically a higher frequency of alternative 3′ splice sites and alternative 5′ splice sites (data not shown). These results were supported with a validation cohort where 91,860 events were detected, being 57 of them significantly different (Supplemental Fig. 2), showing a similar pattern of distinct splicing events depending on *SF3B1* expression levels. Interestingly, exon skipping and alternative 3′ splice site events were over-represented in PDAC samples expressing high SF3B1 levels, while mutually exclusive exons, alternative first exon and alternative last exon events prevailed in tumors expressing low SF3B1 levels (Fig. 2C). Importantly, some of the most pronounced changes were validated in an external PDAC cohort (Supplemental Fig. 3).

KEGG analysis of the genes differentially spliced, depending on *SF3B1* expression, revealed a particularly tight association with the “pancreatic cancer” category (the term with the highest gene ratio i.e., number of hits divided by the total genes of that KEGG term), but also with colorectal cancer and relevant signaling pathways in cancer (Fig. 2D). Moreover, analysis of the genes provided by KEGG and Reactome allowed for identification of a number of key signaling pathways, particularly AKT-related (Fig. 2E).

Further analysis of our RNA-seq data and the PanCancer dataset indicated that *SF3B1* expression levels correlated directly with *KRAS*, *BRCA1*, *BRCA2,* and *HNRNPK* and inversely with *CDKN2A* and *TP53* mRNA levels (Fig. 2F-G). Conversely, *SF3B1* expression did not seem to be associated with the mutational status of key driver genes (*KRAS, CDKN2A*, *SMAD4*, *TP53, BRCA1, BRCA2,* and *HNRNPK*) in the PanCancer PDAC dataset (Supplemental Fig. 4).

### SF3B1 inhibition alters functional features as well as signaling and splicing mechanisms in PDAC cell lines

**Fig. 3:**
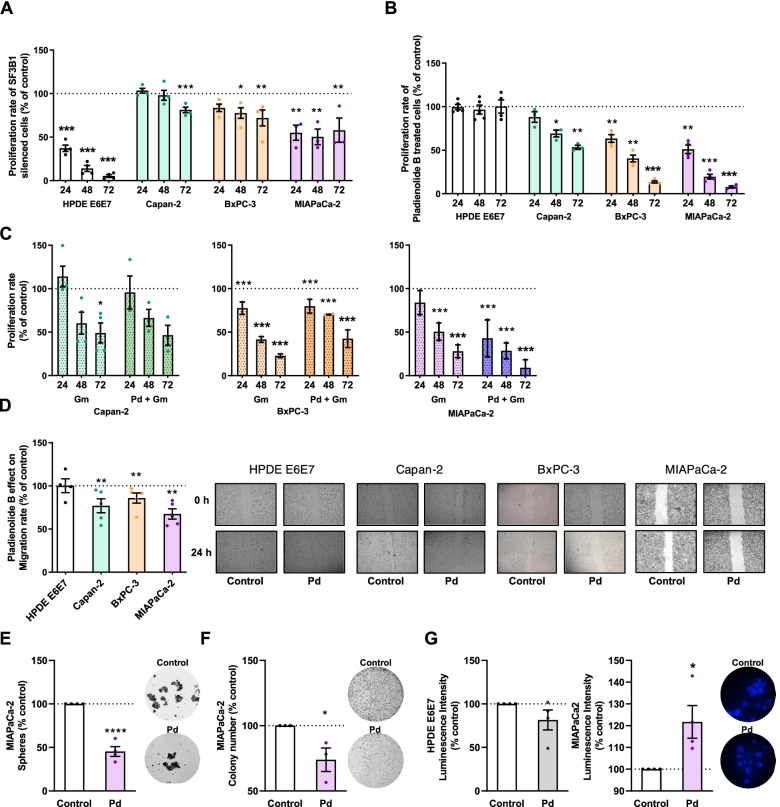
Effect of SF3B1 modulation on PDAC cell lines. **A** Proliferation rates of HPDE E6E7, Capan-2, BxPC-3, and MIAPaCa-2 cell lines after *SF3B1* silencing compared with scramble control-silenced cells (set at 100%; dotted line; *n* = 3–4). **B** Proliferation rates of same cell lines treated with or without (vehicle, set at 100%; dotted line) Pladienolide-B (*n* = 3–5). **C** Gemcitabine (Gm) and Pladienolide-B plus Gemcitabine (Pd + Gm) treated cells compared with vehicle-treated cells (set at 100%; dotted line; *n* = 3–5). **D** Migration rates of HPDE E6E7, Capan-2, BxPC-3 and MIAPaCa-2 cell lines treated with or without (vehicle; set at 100%) Pladienolide-B for 24 h. Representative images of wound closures (*n* = 4). **E** Quantification of sphere formation capacity of MIAPaCa-2 treated with Pladienolide-B or vehicle (control; set at 100%). Representative images of spheres (*n* = 4). **F** Colony formation capacity quantification of MIAPaCa-2 treated with Pladienolide-B or with vehicle (control; set as 100%). Representative images of colony formation (*n* = 3). **G** Apoptosis quantification using Caspase-3/7 assay in HPDE E6E7 and MIAPaCa-2 treated 24 h with Pladienolide-B or vehicle (control; set as 100%) (*n* = 4). Representative images show MIAPaCa-2 nuclear staining with DAPI. Data represents mean ± SEM. Asterisks indicate significant differences (*p < 0.05; **p < 0.01; ***p < 0.001)

To explore the role of SF3B1 in PDAC, we silenced its expression with a specific siRNA or inhibited its function pharmacologically. PDAC cell lines expressed appreciable mRNA levels of *SF3B1* (Supplemental Fig. 5A) that were efficiently silenced (40–80%) in all cells tested (Supplemental Fig. 5B). *SF3B1* silencing time-dependently decreased cell proliferation in PDAC cell lines: well differentiated Capan-2 (less prominently), moderately differentiated BxPC-3, and poorly differentiated MIAPaCa-2 [42], and particularly in the non-tumoral pancreatic cell line HPDE E6E7 (Fig. 3A). We then applied an alternative experimental approach by pharmacologically blunting SF3B1 activity, instead of its expression, using the specific inhibitor Pladienolide-B [11]. Initial screenings in PDAC cell lines using various Pladienolide-B doses led us to select a 1 nM dose for subsequent studies (Supplemental Fig. 5C). Pladienolide-B time-dependently reduced proliferation in all PDAC cell lines (Fig. 3B), in a manner that paralleled their reported degree of aggressiveness. Interestingly, Pladienolide-B did not alter proliferation of non-tumoral HPDE E6E7 cells, suggesting a tumor cell-specific effect. Intriguingly both conditions revealed a distinct cell response, where the anti-proliferative effect of either Pladienolide-B or SFB31 silencing appeared to be associated to changes in intracellular distribution. Specifically, decreases in proliferation were accompanied by an increased proportion of cytoplasmic SF3B1 staining (Supplemental Fig. 6). Comparing the actions of Pladienolide-B and the first-line PDAC chemotherapeutic drug Gemcitabine showed that both drugs exerted comparable effects on all PDAC cell lines tested; however, their combination did not produce an additive inhibitory effect (Fig. 3C).

Pladienolide-B reduced the migration rate of the three PDAC cell lines assessed in a wound-healing assay, while no effect was observed in non-tumoral HPDE E6E7 cells (Fig. 3D). Interestingly, MIAPaCa-2 cells, regarded as the most aggressive and stem-like [43] of the three PDAC cell lines tested, displayed the most pronounced reductions in migration and proliferation in response to Pladienolide-B. Hence, this cell line was selected to further explore the effects of the drug in subsequent stem-associated assays, using non-tumoral HPDE E6E7 cells in parallel, where appropriate. Pladienolide-B reduced by half the sphere formation (i.e., self-renewal) capacity of MIAPaCa-2 cells compared to vehicle-treated cells (Fig. 3E). Likewise, Pladienolide-B inhibited colony formation of MIAPaCa-2 cells with respect to vehicle-treated cells (Fig. 3F). Furthermore, Pladienolide-B increased apoptotis in MIAPaCa-2 cells but not in HPDE E6E7 cells (Fig. 3G).

**Fig. 4:**
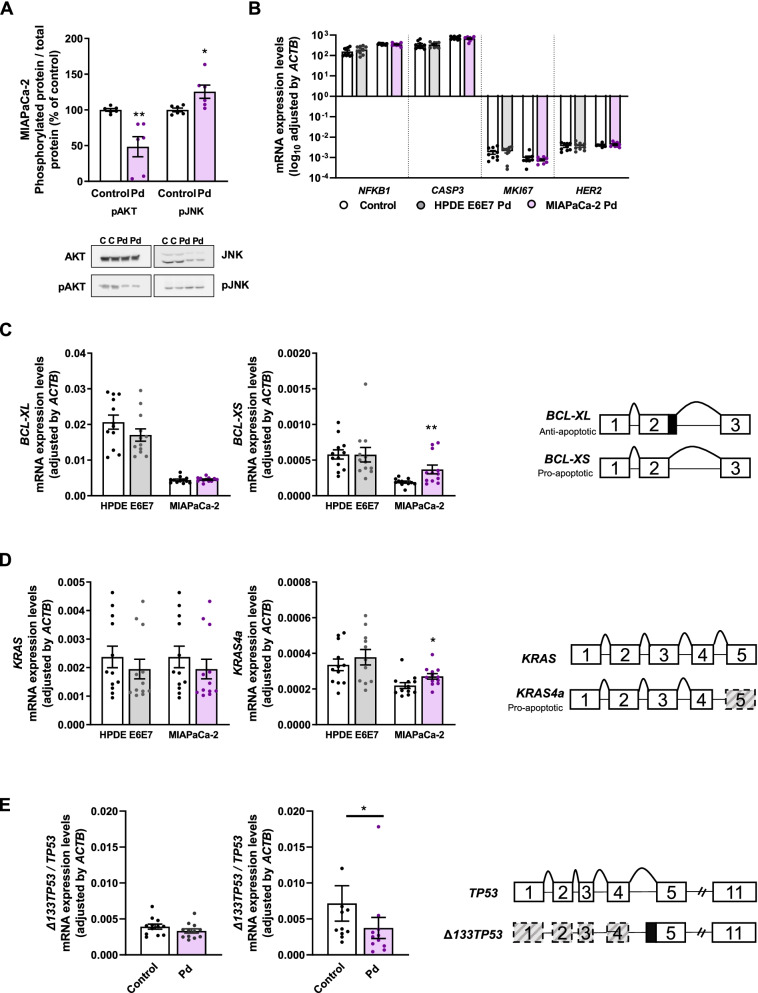
Molecular profile of Pladienolide-B-treated PDAC cell lines. **A** Western-blot analysis of p/tAKT p/tJNK in MIAPaCa-2 cell lines after 24 h Pladienolide-B treatment. Ponceau-stained membranes served as loading control reference (*n* = 6). **B** mRNA levels in malignancy-implicated genes. Values represent the log_10_ of expression compared to vehicle-treated (*n* = 4). **C** mRNA levels of *BCL-XL* and *BCL-XS* in HPDE E6E7 and MIAPaCa-2 cells treated 24 h with or without (vehicle, control) Pladienolide-B (*n* = 4). **D** mRNA expressions of *KRAS* and *KRAS4a* in HPDE E6E7 and MIAPaCa-2 cells treated 24 h with or without (vehicle, control) Pladienolide-B (*n* = 4)*.* **E** Ratio of *Δ133TP53/TP53* mRNA levels in HPDE E6E7 and MIAPaCa-2 cells treated 24 h with or without (vehicle, control) Pladienolide-B (*n* = 4). Gene expression was normalized to *ACTB* expression. Asterisks indicate significant differences (*p < 0.05; **p < 0.01; ***p < 0.001)

To gain mechanistic insights into the observed effects of Pladienolide-B, we explored the activation, expression or splicing of key signaling players/regulatory genes in PDAC cells (Fig. 4). Pladienolide-B decreased AKT and increased JNK phosphorylation in MIAPaCa-2 cells (Fig. 4A) without altering ERK1/2 phosphorylation (not shown). Intriguingly, Pladienolide-B did not influence the expression of genes relevant to tumor biology (apoptosis, proliferation, inflammation) in MIAPaCa-2 cells or in HPDE E6E7 cells, including *NFKB1*, *CASP3*, *MKI67*, and *HER2* (Fig. 4B). Conversely, this drug did modify the expression pattern of splicing-related isoforms of key PDAC-related genes. Thus, Pladienolide-B increased the levels of the pro-apoptotic splice isoform *BCL-XS* but not of the long, anti-apoptotic *BCL-XL* isoform in MIAPaCa-2 cells. Importantly, these effects were not observed in HPDE E6E7 cells (Fig. 4C). Furthermore, while Pladienolide-B did not alter total *KRAS* mRNA levels in HPDE E6E7 or MIAPaCa-2 cells, it augmented the expression of the splice isoform *KRAS4a* only in MIAPaCa-2 cells (Fig. 4D). Pladienolide-B also modulated *TP53* in MIAPaCa-2, but not in HPDE E6E7 cells, by increasing full-length *TP53* expression while blunting its truncated variant *Δ133TP53*, resulting in a decreased *Δ133TP53*/*TP53* ratio (Fig. 4E). Therefore, not only is *SF3B1* overexpressed in PDAC, which may influence the splicing profiles in cancer cells, but the splicing inhibitor Pladienolide-B reduces pivotal functional (proliferation, migration) and stem-associated features (colony- and sphere-formation), likely by altering key signaling and splicing events.

### Pladienolide-B attenuates PDAC stemness functional properties

**Fig. 5:**
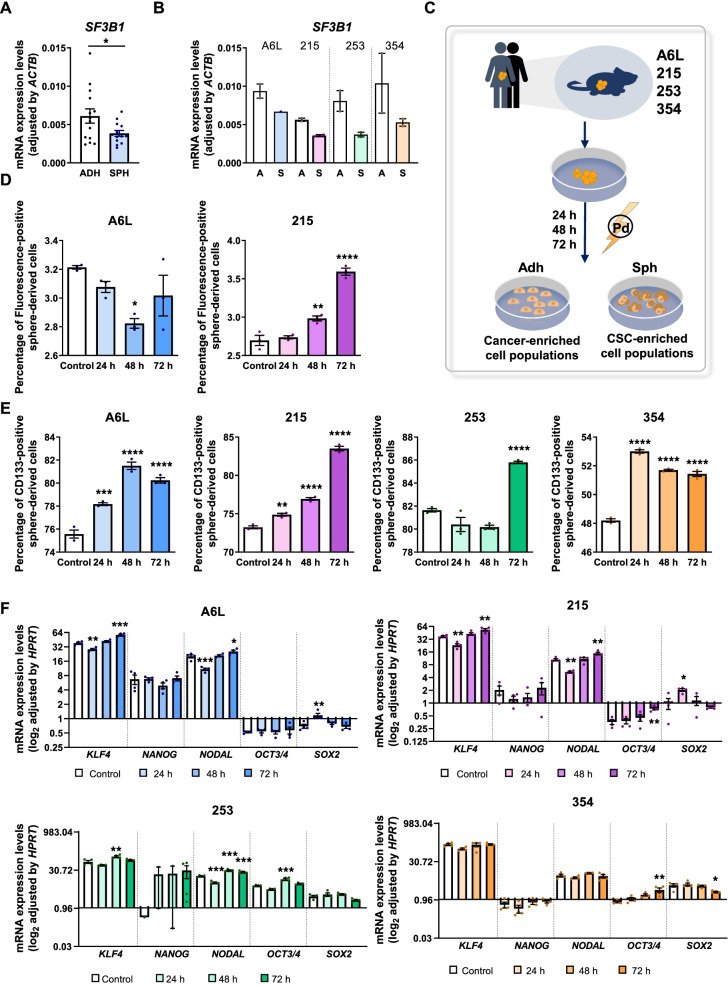
*SF3B1* expression and consequences of its modulation in PDAC CSCs. **A**, **B** *SF3B1* mRNA levels (normalized to *ACTB* expression) in adherent (ADH; A)- vs. sphere (SPH; S)-derived PDX PDAC cells. The SF3B1 mRNA levels are grouped in A or individual in B for the PDX-derived cell set (A6L, 215, 253 and 354) (*n* = 2). **C**. Representative scheme of PDAC PDX-derived cell line generation and treatment with Pladienolide-B. **D**, **E** Quantification of flow cytometric analysis of the percentage of autofluorescent (Fluo) and CD133-positive cells in the indicated PDX-derived in vitro cultures treated with or without (vehicle) 1 nM Pladienolide-B (*n* = 3). **F** Log_2_ mRNA expressions levels of genes implicated in stemness normalized to *HPRT* expression (*n* = 4). Data shown are mean ± SEM. Asterisks indicate significant differences (*p < 0.05; **p < 0.01; ***p < 0.001)

To investigate a possible role for SF3B1 in pancreatic CSCs, we tested four previously characterized human PDX-derived cell lines (i.e., A6L, 215, 253, and 354), which contain bona fide pancreatic CSCs (Fig. 5) [20]. *SF3B1* expression was first evaluated in adherent (ADH) and spheroid (SPH) cell cultures derived from these cell lines, which represent, respectively, cancer- and CSC-enriched cell populations from the corresponding PDXs. While all the tumors analyzed expressed *SF3B1*, levels were lower in spheroid CSC-enriched cultures (both on average and in each line), suggesting that CSCs naturally express less *SF3B1* than their more differentiated counterparts (Fig. 5A, B).

To assess the impact of SF3B1 blockade specifically in CSCs, Pladienolide-B effects on PDX-derived cell lines were tested using multiple stem-related assays (Fig. 5C). First, the levels of autofluorescence and CD133, established pancreatic CSC markers [20], were evaluated in sphere-derived cells from PDX-derived cell lines. Interestingly, Pladienolide-B transiently (at 48 h) reduced autofluorescence in A6L cells while causing an early and sustained (24-72 h) increase in 215 cells, suggesting CSCs enrichment (Fig. 5D; not measured in 354 and 253 cells, which lack autofluorescence). Importantly, in all cell lines Pladienolide-B induced an early (except 253 cells) and sustained enrichment in CD133, again suggesting an enrichment in CSC-marker positive cells (Fig. 5E).

We next evaluated the influence of Pladienolide-B on the expression of CSC stem- or pluripotency-related transcription factors in PDX-derived cell lines, which, except for 354 cells, largely showed comparable response patterns (Fig. 5F). Specifically, *KLF4* and *NODAL* expression displayed a biphasic response in A6L, 215 and 253 cells, whereas *SOX2* expression increased in A6L and 215 cells at 24 h, and decreased at 72 h in 354 cells, and *OCT3/4* displayed disparate responses among the cell lines (Fig. 5F). These data suggests that Pladienolide-B only marginally influences the transcription of stem-associated genes in PDX-derived PDAC cells.

**Fig. 6:**
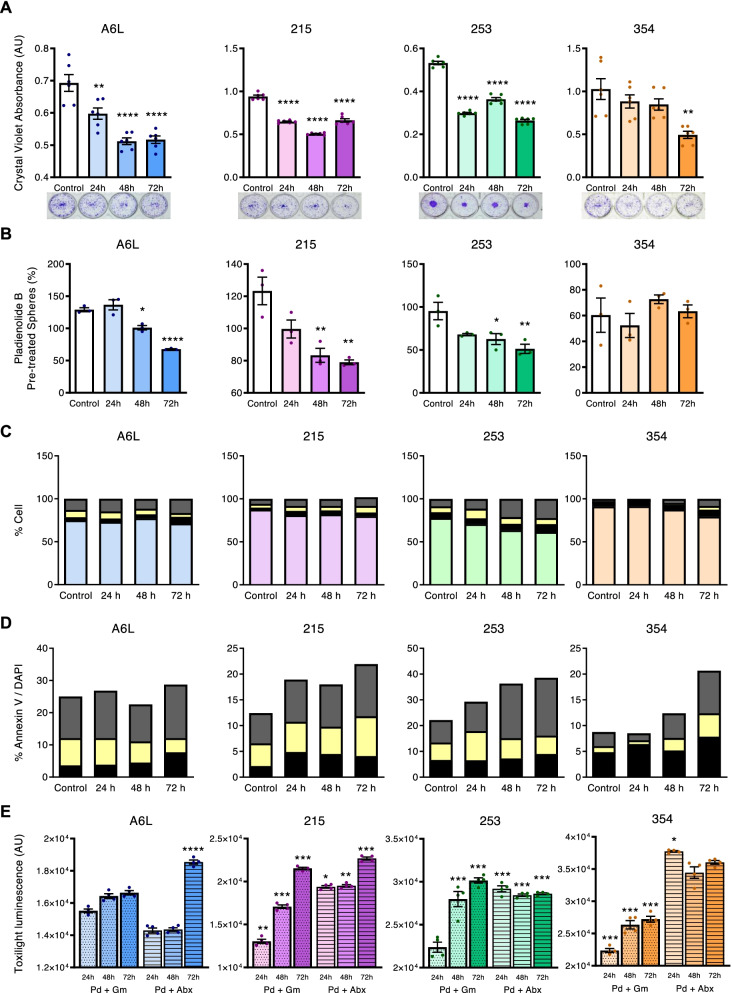
Effect of Pladienolide-B on PDAC CSC functional properties. **A** Colony formation efficiency represented as crystal violet absorbance (AU; arbitrary units) in PDAC PDX-derived cells after Pladienolide-B treatment compared with vehicle-treated cells. Representative images of colony formation (*n* = 6). **B** Sphere formation efficiency (number of spheres/mL) in PDAC PDX-derived cells after Pladienolide-B treatment compared to vehicle-treated cells (*n* = 3). **C**, **D** Quantification of annexin-V staining in Pladienolide-B-treated cells across PDX-derived in vitro cultures compared with vehicle-treated cells. Top and bottom: live cells (blue); dead cells (black); early apoptosis (yellow) and late apoptosis (grey) (*n* = 1). **E** Cell death, measured with the bioluminescence Toxilight assay, after treatment with the indicated compounds in combination with Pladienolide-B (*n* = 4). Data represents mean ± SEM. Asterisks indicate significant differences (*p < 0.05; **p < 0.01; ***p < 0.001)

While the above data could suggest a CSCs enrichment, we examined the functional consequences of Pladienolide-B treatment, by evaluating the capacity of PDX-derived PDAC cell lines to form colonies or spheres. Pladienolide-B reduced the colony-formation capacity of A6L, 215 and 253 cells in a drastic, rapid (24 h) and sustained (72 h) manner, while 354 cells showed a slightly delayed (72 h) response (Fig. 6A). Accordingly, Pladienolide-B clearly reduced the capacity of A6L, 215 and 253 PDX-derived PDAC cell lines to form spheres (Fig. 6B), mimicking the response of MIAPaCa-2 cells treated with Pladienolide-B (Fig. 3E).

The effect of Pladienolide-B on the viability of PDX-derived PDAC cell lines was limited (Fig. 6C), with live cells consistently remaining above 60% in the presence of Pladienolide-B. However, an apparent responsiveness gradient was noticed when apoptosis was assessed, with A6L cells exhibiting higher resistance and 253 cells being more sensitive to Pladienolide-B (Fig. 6D). Early and late apoptotic rates revealed a clear time-dependent trend towards increased late apoptosis, particularly in 354 and 253 cells. Thus, the effects observed in sphere and colony formation may result from Pladienolide-B selectively targeting CSCs.

A hallmark of CSCs is their inherent chemoresistance. Thus, we tested the capacity of Pladienolide-B to sensitize pancreatic CSCs to Gemcitabine or Abraxane, two first-line PDAC treatments. A luminescence-based toxicity assay showed that Pladienolide-B increased the cytotoxic capacity of Gemcitabine and Abraxane, with 215 and 253 cell lines showing the highest cell death increase upon addition of Pladienolide-B compared to Gemcitabine or Abraxane alone (Fig. 6E). As expected, A6L and 354 cells were more resistant. These results demonstrate that SF3B1 is present in PDAC CSCs and that targeting its function with Pladienolide-B cause alterations that reduce key stemness features, decreasing their ability to form colonies and spheres, and enhancing their susceptibility to Gemcitabine or Abraxane.

### Pladienolide-B affects PDAC cell and CSC in vivo tumor formation

**Fig. 7:**
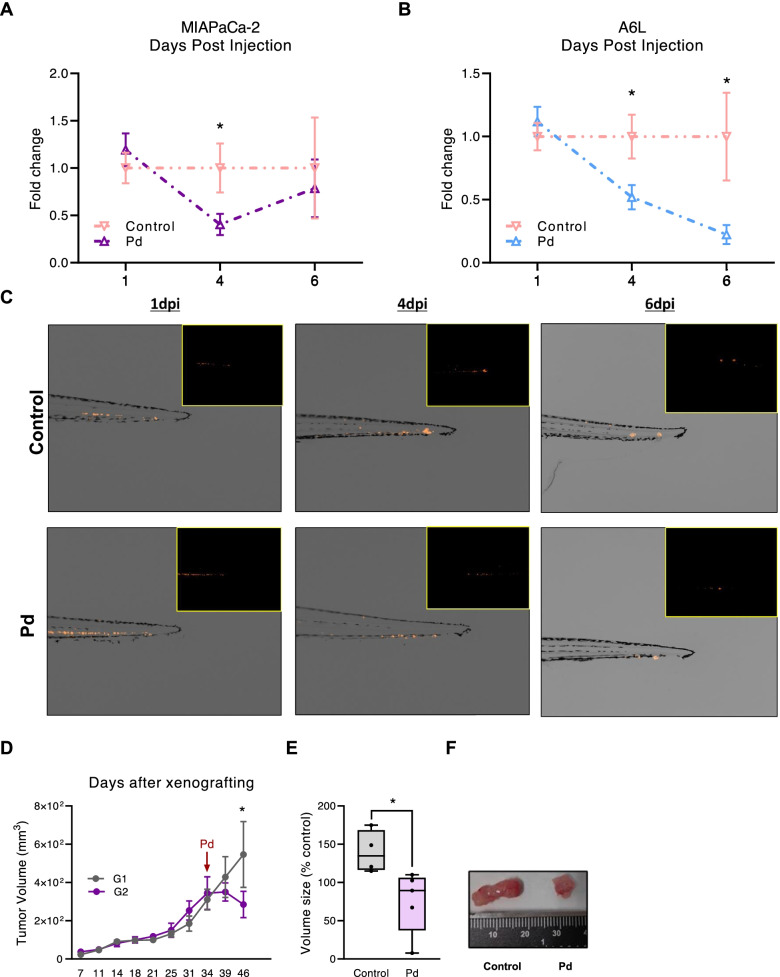
Pladienolide-B reduces malignancy features of PDAC cells and CSCs in vivo. **A**-**B** Fold-change ± SEM of MIAPaCa-2 and A6L h2b-mCherry cells in zebrafish embryos, calculated by measuring the area and the fluorescence intensity. Cells were injected after 24 h of Pladienolide-B (Pd) treatment. Changes in Pd-treated cells were compared to control at indicated days post injection (dpi). **C** Representative images of Control- and Pd-treated A6L-zebrafish xenografts at indicated dpi. **D** Tumor volume (mm^3^) of MIAPaCa-2-xenografts at indicated days after xenografting. Red arrow indicates Pladienolide-B injection. G1, control-treated mice, G2, Pd-treated mice. **E** MIAPaCa-2 xenograft tumor volumes, expressed as % ratio, extracted from Control- (*n *= 4) and Pd-treated (*n* = 5) mice at time of euthanasia (d49 after xenografting). **F** Pictures of paired Control- and Pd-treated tumors. Asterisks indicate significant differences (*p < 0.05; **p < 0.01; ***p < 0.001)

To test whether the inhibitory effects exerted by Pladienolide-B in vitro in PDAC cell lines and PDX-derived cell lines could also be observed in vivo, we employed two complementary preclinical models. First, PDAC cells were intravenously injected in zebrafish (an adequate system for real-time tracking of CSC-mediated early metastasis and tumor formation) [40]. Specifically, MIAPaCa-2 and A6L cells, stably infected with an mCherry-H2B expressing lentivirus, were treated in vitro with Pladienolide-B or vehicle prior to microinjection into circulation [44]. While the inhibitory actions of Pladienolide-B pre-treatment were not observed at 1-dpi, they became evident thereafter. Embryos injected with pre-treated MIAPaCa-2-mCherry-H2B cells showed a marked reduction in cell dissemination at 4-dpi, while those injected with pre-treated A6L-mCherry-H2B PDX-derived cells showed a drastic reduction in tumor cell dissemination and growth at 4-dpi, which was further enhanced at-6 dpi (Fig. 7A, B; representative images, 7C).

Finally, tumor xenografts were generated in nude mice (subcutaneous injections in both flanks, paired tumors) with MIAPaCa-2 cells. After tumors reached approximately 100mm^3^ (34-dpi), diluent control (G1) or Pladienolide-B (G2) were injected intratumorally and tumor growth was evaluated over the next 12 days. In line with our in vitro and zebrafish results, a single Pladienolide-B dose was sufficient to significantly reduce tumor growth (Fig. 7D). Likewise, appreciable differences in tumor weight and size were observed when tumors were resected (Fig. 7E-F). Examination of the presence of necrosis in the tissue of xenograft tumors did not reveal any appreciable difference between diluent- and Pladienolide-B-treated tumors. In contrast, Ki67 analysis showed a downward but non-significant trend in Pladienolide-B treated xenografts (Supplemental Fig. 7). Moreover, in line with previous results in cell lines, particularly MIAPaCa-2 cells, we observed similar trends in the alternative splicing of the genes examined (Supplemental Fig. 8).

Thus, the use of two different animal models indicates that Pladienolide-B treatment can reduce early metastasis and tumor cell proliferation of PDAC cells as well as retract tumor growth of PDAC xenografts, highlighting the potential of Pladienolide-B to treat PDAC .

## Discussion

In the present study, we not only show that the pivotal splicing machinery component SF3B1 is overexpressed in PDAC, but it can be targeted by Pladienolide-B, which causes antioncogenic effects in both cancer cells and CSCs, paving the way to develop new treatment strategies for this deadly cancer.

SF3B1 dysfunction, through mutation [7, 13, 14] or altered expression [16, 17], is known to increase oncogenic features in various cancers including PDAC [15, 34]. We now show that *SF3B1* is overexpressed in PDAC as compared to its surrounding tissue (our samples) or healthy pancreatic tissue (E-MTAB-1791-cohort) [22]. Importantly, IHC analysis confirmed its overexpression in tumor cell nuclei. Moreover, *SF3B1* levels were associated to relevant clinical parameters, suggesting a potential pathological relevance linked to its dysregulation. These results are in line with our recent studies in prostate cancer [16] and hepatocarcinoma [17], and other studies in chronic lymphocytic leukemia or endometrial and breast cancers [38, 45, 46], collectively reinforcing the growing view that this splicing factor is heavily altered in cancer. Accordingly, we asked whether *SF3B1* expression could be associated with or even contribute to PDAC pathophysiology.

To answer this question, we first biocomputationally explored the link between *SF3B1* expression and its primary regulatory endpoint, alternative splicing, and found that PDAC with high or low *SF3B1* expression displayed strikingly distinct global splicing patterns. Interestingly, high *SF3B1* levels correlated with higher usage of alternative 3′ splice sites, resembling common alterations in *SF3B1*-mutated cancers [7], and with elevated exon skipping, which has been linked to PDAC and to *SF3B1*-mutation in myelodysplastic syndromes [47] and *C. elegans* models [48]. Conversely, low *SF3B1* expression was associated with elevated frequency of splicing events not particularly linked to mutant *SF3B1* malignancies [7, 13, 14]. These findings suggest that, as a key splicing player, *SF3B1* may influence the global splicing pattern in PDAC, thereby potentially having pathological implications [6–8]. Reactome analysis revealed that the spliced genes associated to *SF3B1* expression were tightly coupled to both PDAC and its central AKT-signaling pathway [49, 50]. Further analyses revealed that *SF3B1* expression levels were linked to key PDAC molecular features, including direct correlations with *KRAS*, *BRCA1*, *BRCA2* and *HNRNPK* expression and inverse correlations with *CDKN2A* and *TP53* expression. This multifaceted association of *SF3B1* expression with global splicing and expression levels of key PDAC genes converges with recent data linking PDAC malignancy to splicing dysregulation [11], strongly suggesting that *SF3B1* overexpression in PDAC, like in prostate cancer [16] and hepatocarcinoma [17], may have pathological consequences.

To interrogate *SF3B1* function in PDAC, we first silenced its expression in normal pancreatic (HPDE E6E7) and PDAC cell lines (Capan-2, BxPC-3, MIAPaCa-2), where a time-dependent decrease in cell proliferation, particularly in HPDE E6E7 cells, was observed. These results agree with findings in mice showing that *Sf3b1* homozygote deletion is embryonic lethal [51], and in cancer cell lines, where *SF3B1* copy number loss represents a vulnerability, suggesting its essential role [52]. Notably, in keeping with this latter study [52] and our previous work [16], pharmacological inhibition of SF3B1 function with Pladienolide-B markedly decreased PDAC cell proliferation without affecting HPDE E6E7 cells, unveiling a difference in cell function when targeting SF3B1 with inhibitors vs. modulating its expression genetically [52]. Interestingly, Pladienolide-B’s antiproliferative action was comparable to that of Gemcitabine, although their combination did not potentiate each other (at least in established cell lines), suggesting shared mechanism(s) of action(s). Notably, Pladienolide-B not only affected proliferation but also inhibited cell migration and sphere and colony formation, while enhancing cell apoptosis. These findings underscore the promising anticancer capacity of Pladienolide-B in PDAC cells and expands upon the cancers wherein pharmacologically targeting SF3B1 exerts anticancer actions.

The mechanisms underpinning Pladienolide-B actions in PDAC likely involves alteration of key signals, as suggested by the concomitant reduction of pAKT and increase in pJNK, two critical kinases that regulate vital cellular processes in PDAC and other cancers [49, 50, 53–55]. In PDAC, AKT overexpression is a common feature closely linked to cell plasticity [49, 50], which also seems to be linked to JNK, that could act as a tumor suppressor [53–55]. Pladienolide-B inhibition of SF3B1 function not only modulated signaling cascades in tumor cells but also altered splicing of molecules crucial in PDAC, such as *BCL-X*, *KRAS* and *TP53*, favoring the balance of the more pro-apoptotic and/or antioncogenic variants. Specifically, Pladienolide-B increased the *BCL2L1* isoform *BCL-XS*, which binds to and inhibits its antiapoptotic variant *BCL-XL* and *BCL2* itself, thereby promoting the release of proapoptotic BAK [56]. Likewise, Pladienolide-B treatment increased the proapoptotic variant *KRAS4a* [57] without altering full-length *KRAS4*, and reduced the proportion *Δ133TP53/TP53*, likely fostering apoptosis, inasmuch as *Δ133TP53* inhibits p53 [58]. These results provide experimental support that SF3B1 directly impacts relevant splicing phenomena in PDAC, which was prompted by the aforementioned association of *SF3B1* expression levels with distinct splicing event profiles. Hence, Pladienolide-B would act on PDAC cancer cells by altering both, key signaling pathways and splicing mechanisms.

Having established the antioncogenic actions of Pladienolide-B in PDAC cells, we next interrogated its potential effects on CSCs, a unique cell subset increasingly recognized as a relevant player in PDAC maintenance, chemoresistance, disease relapse and metastasis [18]. Although recent evidence suggests a splicing machinery dysregulation in PDAC CSCs [21], *SF3B1*’s role in these cells is still unknown. In our PDX-derived CSC-enriched models, *SF3B1* expression levels were appreciable but lower than in cancer cell lines. Correspondingly, CSCs presented lower protein levels for related splicing machinery components (SF3B2, SRSF1, hnRNPs) compared to PDAC Panc1 cells, which may be linked to maintenance of the CSC “dedifferentiated” state [21]. Intriguingly, Pladienolide-B appeared to preferentially target cell survival and apoptosis in cancer cells over CSCs in PDX-derived cultures, potentially suggesting CSCs drug resistance. However, further examination revealed that Pladienolide-B was able to affect other crucial CSC features, altering pluripotency-related gene expression (e.g., *KLF4*, *NODAL* and *SOX2*) and decreasing sphere- and colony-formation capacity, which reflect a loss in self-renewal and stem properties. More importantly, Pladienolide-B also reduced CSC chemoresistance, as its combination with chemotherapeutic drugs (e.g., Gemcitabine or Abraxane) markedly increased toxicity.

We were surprised that while Pladienolide-B inhibited CSC functional properties (self-renewal, chemoresistance, tumorigenicity), CSC marker-positive populations increased. We hypothesize that the latter could result from cancer cell plasticity. Indeed, non-CSC hybrid/transient cells can dedifferentiate and convert into CSCs when the CSC compartment is compromised [59, 60]. Since Pladienolide-B enhanced apoptosis in all PDAC PDX-derived cultures concomitant with a decrease in CSC functional phenotypes, we can only assume that CSC-negative cells were attempting to replenish the CSC pool, resulting in increased autofluorescent- and CD133-positive cells. While confirming this hypothesis requires further studies, the fact that Pladienolide-B treatment reduces functional CSC properties is proof enough that the CSC compartment is affected by SF3B1 modulation.

As proof-of-concept that Pladienolide-B’s antioncogenic effects in vitro are clinically translatable, we tested its actions in vivo in two previously validated preclinical models [17, 40]. Indeed, Pladienolide-B pretreatment of MIAPaCa-2 cells or CSCs blunted their capacity to migrate and proliferate in a zebrafish model, supporting the anti-invasive and anti-metastatic effects of Pladienolide-B. Moreover, Pladienolide-B prevented tumor growth in mice with MIAPaCa-2 tumor xenografts, which did not present systemic or histological problems (metastasis, necrosis), in keeping with our data in prostate cancer [16, 17] and hepatocarcinoma [17], and the antitumoral actions of spliceosome-targeted drugs in PDAC mouse models [11]. Thus, these two distinct models provide suggestive evidence that by inhibiting SF3B1 with Pladienolide-B, the oncogenic properties of both PDAC cells and CSCs are reduced in vivo.

## Conclusion

In summary, our findings reveal that SF3B1 is overexpressed in human PDAC, where its levels associate with key clinical (lymph node stage), histological (grade), and molecular (e.g., splicing alterations) features. Furthermore, targeting SF3B1 function with Pladienolide-B reduces multiple cancer features in PDAC cells (proliferation, migration, and colony and sphere formation) by altering relevant signaling pathways and splicing events. Importantly, Pladienolide-B treatment reduces CSCs stemness, making CSCs more sensitive to chemotherapy treatment. Finally, this drug’s anti-tumoral and anti-CSC effects were also observed in two distinct in vivo preclinical models, xenografted zebrafish and mice. We conclude that SF3B1 overexpression represents a therapeutic vulnerability in PDAC that enables the targeting of splicing with Pladienolide-B not only in cancer cells but also in CSCs, which opens up novel therapeutic avenues for this lethal cancer.

## Supplementary Information

**Additional file 1.**

## Data Availability

The datasets used and/or analyzed during the current study are available from the corresponding author upon reasonable request.

## Abbreviations

ADH: Adherent
BPE: Bovine pituitary extract
CHT: Caudal hematopoietic tissue
CSC: Cancer stem cells
DAPI: 4′,6-diamidino-2-phenylindole
DPI: Days post injection
EGF: Epidermal growth factor
FBS: Fetal bovine serum
FFPE: Formalin-fixed paraffin-embedded
HS: Horse serum
IHC: Immunohistochemistry
PBS: Phosphate buffered saline
Pd: Pladienolide-B
PDAC: Pancreatic ductal adenocarcinoma
PFA: Paraformaldehyde
qPCR: Quantitative polymerase chain reaction
RT: Room temperature
SF3B1: Splicing factor 3B subunit 1
siRNA: Small interference RNA
snRNP: Small nuclear RNA
SPH: Sphere
