# Supplementary material for: Dysregulated splicing factor SF3B1 unveils a dual therapeutic vulnerability to target pancreatic cancer cells and cancer stem cells with an anti-splicing drug
Source: J Exp Clin Cancer Res. 2021 Dec 2;40:382. doi: 10.1186/s13046-021-02153-9 (PMC8638119; doi:10.1186/s13046-021-02153-9)
Supplement: Supplementary file 1 — Additional file 1. [file 13046_2021_2153_MOESM1_ESM.pdf]

Graphical Abstract

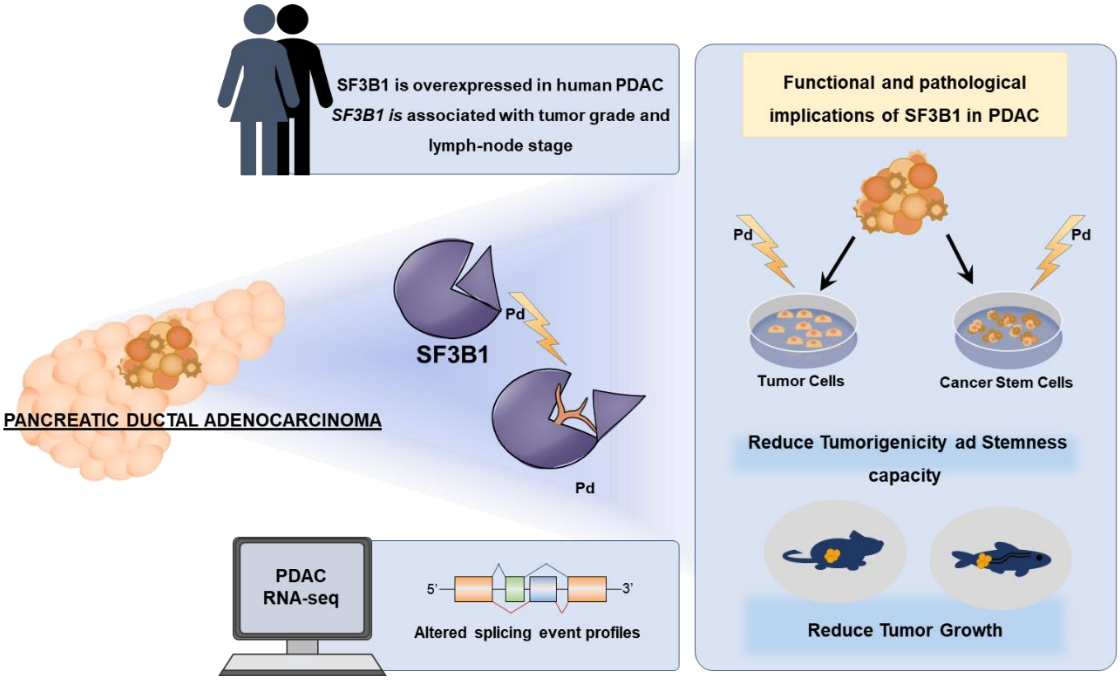

The process that matures mRNA, splicing, is altered in pancreatic cancer, enhancing its malignancy. By targeting the splicing-factor SF3B1, Pladienolide-B inhibits pancreatic cancer cells and cancer stem cells, unveiling a novel approach to treat pancreatic cancer.

Supplemental Table 1. Additional clinical characteristics of the patients

Characteristic		Samples n=75 (100%)
**Smoker**	Yes	28 (37.3)
	Former smoker	11 (14.7)
**Drinker**		50 (66.7)
**Body Mass Index**	< 20	10 (13.04)
	20 – 25	45 (60.87)
	25 – 30	16 (21.74)
	> 30	3 (4.35)
**Diabetes**	Type 1 Diabetes Mellitus	3 (4.2)
	Type 2 Diabetes Mellitus	23 (30.6)
**Arterial Hypertension**		44 (58.6)
**Hypercholesterolemia**		44 (58)
**Prior Acute Pancreatitis**		7 (9.3)
**Family Background**	Pancreatic cancer	1 (1.3)
	Other cancers	2 (2.7)
**Perineural Invasion**		56 (74.7)
**Linfovascular Invasion**	Small blood vessel	22 (29.3)
	Big blood vessel	20 (26.7)
**Grading**	Grade X	2 (2.7)
	Grade 1	15 (20)
	Grade 2	43 (57.3)
	Grade 3	8 (10.7)
	Grade 4	1 (1.3)

Supplemental Table 2. Clinicopathological characteristics of 94 pancreatic adenocarcinoma patients profiled by RNASeq

**Characteristic**	**Samples**<br>**n=94 (100%)**
**Age**	Median 67 (range 37-86)
**Sex**	
Female	46 (48.9)
Male	48 (51.1)
**T stage**	
T1	1 (1.1)
T2	2 (2.1)
T3	91 (96.8)
T4	0 (0)
**N stage**	
N0	12 (12.8)
N1	82 (87.2)
**M stage**	
M0	91 (96.8)
M1	3 (3.2)
**Grading**	
G1	4 (4.3)
G2	60 (63.8)
G3	30 (31.9)
**Vascular invasion**	
Yes	10 (10.6)
No	84 (89.4)
**Perineural invasion**	
Yes	89 (94.7)
No	5 (5.3)
**Fat invasion**	
Yes	85 (90.4)
No	9 (9.6)

Supplemental Table 3. Specific primers for human transcripts used in this study.

	Sense Primer Sequence	Antisense Primer Sequence
ACTB	ACTCTTCCAGCCTTCCTTCCT	CAGTGATCTCCTTCTGCATCCT
GAPDH	AATCCCATCACCATCTTCCA	AAATGAGCCCCAGCCTTC
HPRT1	CTGAGGATTTGGAAAGGGTGT	TAATCCAGCAGGTCAGCAAAG
SF3B1	CAGTTCCGTCTGTGTGTTCG	GCTGCCTTCTTGCCTTGA
KRAS	CTTGGATATTCTCGACACAGCA	AAAGAAAGCCCTCCCCAGT
KRAS4A	ACAGAGAGTGGAGGATGCTTTTT	AGCCAGGAGTCTTTTCTTCTTTG
BCL-XL	GATGGCCACTTACCTGAATGA	TGCTGCATTGTTCCCATAGA
BCL-XS	GAGCTTTGAACAGGATACTTTTGTG	GAAGAGTGAGCCCAGCAGAA
TP53	AAGGAAATTTGCGTGTGGAG	CCAGTGTGATGATGGTGAGG
TP53Δ133	ACTCTGTCTCCTTCCTTACAG	GTGTGGAATCAACCCACAGCT
NFKB1	CCTGTCCTTTCTCATCCCATC	TGCCAGAGTTTCGGTTCACT
CASP3	GTCTCAATGCCACAGTCCAGT	TTTTTCAGAGGGGATCGTTG
MIK67	GACATCCGTATCCAGCTTCCT	GCCGTACAGGCTCATCAATAAC
ERB2	CTGTGTTCCATCCTCTGCTG	TGCCTGTCCCTACAACTACCTT
KLF4	ACCCACACAGGTGAGAAACC	ATGTGTAAGGCGAGGTGGTC
NANOG	TGAACCTCAGCTACAAACAGGTG	AACTGCATGCAGGACTGCAGAG
NODAL	AGCATGGTTTTGGAGGTGAC	CCTGCGAGAGGTTGGAGTAG
OSCT3/4	CTTGCTGCAGAAGTGGGTGGAGGAA	CTGCAGTGTGGGTTTCGGGCA
SOX2	AGAACCCCAAGATGCACAAC	CGGGGCCGGTATTTATAATC

Supplemental Table 4. Specific data for human transcript variants primers used in this study.

	Ensembl id	Gene name	SUPPA_name	dPSI	p-Value	Forward	Reverse
1_AF	ENSG000001531 87	*HNRNPU*	ENSG00000153187,20; AF:chr1:244862730-244863617:244863653: 244862730-244863674:244864091:-	0,484 372	0,0224 8	AGCTAGGAGAG GAGAACGGG	CGAGCTCATCTTCCC CTTCC
2_AF	ENSG000001720 71	*EIF2AK3*	ENSG00000172071,14; AF:chr2:88613853-88624746:88624929:88 613853-88626967:88627464:-	0,484 871	0,0119 9	GCTCCCACCTCA GCGAC	GTCTCATCGTCTGGTT CCGG
3_A3	ENSG000001640 39	*BDH2*	ENSG00000164039,15; A3:chr4:103092718-103095203:103092696-103095203:-	0,488 784	0,0209 8	ACCCGGTGCCT CTTGTTTTA	AACTTCATTGGCAAAC TGATCAA
4_A5	ENSG000002240 32	*NA*	ENSG00000224032,7; A5:chr5:112161295-112161703:112160991-112161703:+	0,492 791	0,0339 7	CATCGACTATGC CAGGGAGT	GCAGGGCAAGCATAA AGTCA
5_AF	ENSG000001025 54	*KLF5*	ENSG00000102554,14; AF:chr13:73054976:73 055158-73061861:73059005:73 059588-73061861:+	0,493 420	0,0050 0	CGCTTGGCCTAT AACTTGGT	TGGAGAGACTGGGAT TGCTT
6_MX	ENSG000001403 91	*TSPAN3*	ENSG00000140391,15; MX:chr15:77054279-77055789:77055863-77070892:77054279-77056064:77056255-77070892:-	0,493 740	0,0269 7	CCGTGCTGGTC TTTCTCAAC	TCCCAAAACCACTACA ACAACT
7_RI	ENSG000001033 63	*ELOB*	ENSG00000103363,15; RI:chr16:2771414:2771 604-2771994:2772102:-	0,506 647	0,0037 5	GAGACCCTGGC TGAGAACTT	GTGTCTCTCCCAGTC CTTCC
8_AF	ENSG000001717 29	*TMEM51*	ENSG00000171729,14; AF:chr1:15152532:151 52784-15214895:15153733:15 153954-15214895:+	0,518 320	0,0269 7	GAACTTCAAAGG GCTGGACG	GCTCCCATTCCCTCTC TGAG
9_AF	ENSG000001270 22	*CANX*	ENSG00000127022,15; AF:chr5:179698417:17 9698603-179705679:179698988: 179699102-179705679:+	0,519 071	0,0158 2	TCGGACTCCTAC CCCTTTTG	GTTCCTGGAGCCGAG ACTT
10_A5	ENSG000001063 99	*RPA3*	ENSG00000106399,11; A5:chr7:7639144-7640320:7639144-7640723:-	0,521 667	0,0124 9	TTTCAGAGACAG CGCGATTG	ATTTCTCGGCACCAAT CAGC
11_A5	ENSG000002037 60	*CENPW*	ENSG00000203760,8; A5:chr6:126340444-126346205:126340399-126346205:+	0,526 509	0,0094 9	GAGATTCCATCC CTTCTCGG	GCCAGTACATGCTCC TTGTT
12_SE	ENSG000001408 48	*CPNE2*	ENSG00000140848,17; SE:chr16:57092790-57093950:57094091-57110708:+	0,528 395	0,0494 5	TGAAGGCGTGG TTTTATGGC	GTAGGTTCTGGCCAC TCACT
13_AF	ENSG000001647 13	*BRI3*	ENSG00000164713,10; AF:chr7:98252379:982 52528-98282351:98281686:98 281937-98282351:+	0,530 920	0,0199 8	AGTTGCCGCGTT CTCTCT	TATCCCCAGGTACCC TCTCC
14_AF	ENSG000001601 79	*ABCG1*	ENSG00000160179,18; AF:chr21:42216077:42 216188-42225671:42219118:42 219304-42225671:+	0,533 905	0,0149 9	TTCACCTTCCGC CATGATCA	ATTCAGCAGGTCCGT CTCAG

15_AL	ENSG000001977 56	*RPL37A*	ENSG00000197756,10; AL:chr2:216500031-216501341:216501462:216500031-216529270:216529454:+	0,541 604	0,0139 9	ATGAAGAGACG AGCTGTGGG	AGCGTTGCATTTGGT CCATT
16_A3	ENSG000002459 10	*NA*	ENSG00000245910,8; A3:chr8:66922725-66925437:66922392-66925437:-	0,542 885	0,0224 8	CGAAGAGCCGT TAGTCATGC	ATACATGCCGCGTGA TCCTA
17_AF	ENSG000001235 62	*MORF4L2*	ENSG00000123562,17; AF:chrX:103685260-103686631:103686705:103685260-103687989:103688064:-	0,547 206	0,0139 9	TGCTTGCTTGGA GATCAGGA	CAGGGAAGGTTCTGC AATCA
18_SE	ENSG000001356 78	*CPM*	ENSG00000135678,12; SE:chr12:68871956-68885792:68885885-68932678:-	0,551 704	0,0419 6	GCTGCGCTGGA TTTCAACTA	CCCACAACAAGAACC CACAG
19_A3	ENSG000001705 84	*NUDCD2*	ENSG00000170584,11; A3:chr5:163457080-163457562:163457005-163457562:-	0,556 731	0,0239 8	AGCTGATGAGG GAACATGGA	GAAGTCCAACAATTTG CTGCA
20_A5	ENSG000001417 50	*STAC2*	ENSG00000141750,7; A5:chr17:39217173-39217867:39217173-39218069:-	0,557 282	0,0324 7	CTCCCCATGCC CAGTCC	CATGTTCCTGGAAGC TGTGC
21_SE	ENSG000001368 95	*GARNL3*	ENSG00000136895,19; SE:chr9:127385145-127387193:127387331-127388904:+	0,557 902	0,0279 7	CTGCAGCTGTG AATGAGGTC	GTCTGAGGAGAATTT CGGGC
22_A3	ENSG000001627 04	*ARPC5*	ENSG00000162704,16; A3:chr1:183627594-183630461:183623471-183630461:-	0,560 535	0,0059 9	AGGGTCCATTGT TCGTGTCT	TCCCGAGGCAGATAA TCCAC
23_AF	ENSG000001982 42	*RPL23A*	ENSG00000198242,14; AF:chr17:28719985:28720030-28720707:28720299:28720328-28720707:+	0,568 048	0,0045 0	ACCCTTTTCACA AGATGGCG	GTGACGTGCGGATCT TCTTC
24_AF	ENSG000000754 26	*FOSL2*	ENSG00000075426,12; AF:chr2:28392802:28392836-28404107:28395511:28395776-28404107:+	0,568 878	0,0233 1	CAGGGCTGGAG AATAAAGAGT	AGGGTATGGGTTGGA CATGG
25_SE	ENSG000002298 33	*PET100*	ENSG00000229833,10; SE:chr19:7630846-7631232:7631321-7631473:+	0,570 591	0,0040 0	TAAGGAACCGA GAGCAGAGG	TGGGATCCGCTTCAC TCTTC
26_AF	ENSG000002400 65	*PSMB9*	ENSG00000240065,8; AF:chr6:32844136:32844274-32856138:32854148:32854289-32856138:+	0,570 656	0,0457 0	GCTTCTCTGCTC TCCCGTTA	ATCAGAACCCATCACA ACGC
27_AF	ENSG000000115 66	*MAP4K3*	ENSG00000011566,15; AF:chr2:39378123-39379752:39379809:39378123-39436892:39437301:-	0,577 151	0,0139 9	CAGGAGGACTT CGAGCTGAT	CATTCCGTGCCTTGTA GACG
28_SE	ENSG000001162 09	*TMEM59*	ENSG00000116209,12; SE:chr1:54047372-54048650:54048682-54053000:-	0,583 466	0,0104 9	AGAAGAGGAGT TGTACGCATG	TCCATCATCCACAAAC TGACA
29_SE	ENSG000001563 81	*ANKRD9*	ENSG00000156381,9; SE:chr14:102508549-102508641:102508846-102509529:-	0,588 917	0,0219 8	ACCCTCCTATCT CCTCCCAG	GTTCCGGGGATGTGG GTATA
30_AF	ENSG000001532 50	*RBMS1*	ENSG00000153250,20; AF:chr2:160367391-160416138:160416178:160367391-160493289:160493807:-	0,591 632	0,0149 9	AGCTTCATGGG CAAAGTGTG	AGGTTCGTTTTGCTGA GCTG

31_AL	ENSG000001057 50	*ZNF85*	ENSG00000105750,15; AL:chr19:20935047-20942801:20943977:20935047-20945499:20945709:+	0,598 403	0,0479 5	TCTGGAGCAAG GGAAAGAGG	GGAGAATTGCTTGAA CCCAGG
32_AF	ENSG000001133 87	*SUB1*	ENSG00000113387,12; AF:chr5:32531633:32531916-32588512:32585559:32585625-32588512:+	0,604 633	0,0104 9	ACAGTCCTCTTA GTGCACCA	CTCCACCATCTCTGA GCTGT
33_SE	ENSG000001709 06	*NDUFA3*	ENSG00000170906,16; SE:chr19:54106011-54106353:54106446-54106811:+	0,605 968	0,0040 0	CCGTCATGATCA ACAAGGCC	ACAATCCTGGCTAACA CGGT
34_AF	ENSG000000914 90	*SEL1L3*	ENSG00000091490,11; AF:chr4:25847864-25860601:25860631:25847864-25863434:25863537:-	0,608 459	0,0349 7	CGCAAAAGGGA ATGTTGTACC	GCTCAGCTTTGGGTA TCACTG
35_AL	ENSG000001270 22	*CANX*	ENSG00000127022,15; AL:chr5:179726759-179728591:179728907:179726759-179729360:179729649:+	0,619 790	0,0142 4	AGAAGATGGTG GCACTGTCA	TCTATTCCGGAGCTCA CGTG
36_A3	ENSG000002309 89	*HSBP1*	ENSG00000230989,7; A3:chr16:83809425-83811421:83809425-83817786:+	0,638 175	0,0079 9	AAACAAGATACC TGCCACGC	TCGATCTTCTCTTGGC TTGGA
37_AF	ENSG000002413 43	*RPL36A*	ENSG00000241343,10; AF:chrX:101391016:101391046-101391459:101391202:101391235-101391459:+	0,643 030	0,0074 9	GCAAGCATGGTT AACGTCCC	AATCCTTGCCCTTCTT GTACTG
38_SE	ENSG000002413 43	*RPL36A*	ENSG00000241343,10; SE:chrX:101391822-101392050:101392095-101395335:+	0,655 676	0,0059 9	GAAAGCGGCGT TATGACAGG	TAGGAAAGCAGGGCA CATTC
39_A5	ENSG000001828 99	*RPL35A*	ENSG00000182899,17; A5:chr3:197951328-197954003:197951311-197954003:+	0,664 481	0,0119 9	GTAAGTTTATGA CACTGCAACACA	CATGGGCCCGAGTTA CTTTT
40_AF	ENSG000001600 14	*CALM3*	ENSG00000160014,17; AF:chr19:46601074:46601176-46605827:46601276:46601437-46605827:+	0,671 171	0,0279 7	GCGGCGAGGGA AAGTAGT	GAATCTCTCGTCCCC ACCC
41_AF	ENSG000001667 41	*NNMT*	ENSG00000166741,7; AF:chr11:114295825:114296710-114297951:114297363:114297398-114297951:+	0,676 463	0,0030 0	CCTAGACGGTG TGAAGGGAG	GTCAGTGACGACGAT CTCCT
42_A3	ENSG000002238 65	*HLA-DPB1*	ENSG00000223865,11; A3:chr6:33080534-33080672:33080534-33080689:+	0,684 339	0,0000 0	TTCTCTCTCTGC GTGGTGAG	TTCTCGAGTTCTGTGG TCCT
43_AF	ENSG000001850 88	*RPS27L*	ENSG00000185088,14; AF:chr15:63156521-63157400:63157477:63156521-63157916:63158021:-	0,697 218	0,0000 0	AAGCAAACCTAA GGCACAGC	TGGACTTTGTACTAGG CGTTTC
44_A5	ENSG000001126 95	*COX7A2*	ENSG00000112695,11; A5:chr6:75237988-75240277:75237988-75240301:-	0,702 383	0,0050 0	AAAGGGTGGGG TAGCTGATG	CTTCAAACATTCCAAA GGCCTT
45_AL	ENSG000001977 56	*RPL37A*	ENSG00000197756,10; AL:chr2:216500031-216501341:216504086:216500031-216529270:216529454:+	0,755 854	0,0139 9	ATGAAGAGACG AGCTGTGGG	AGCGTTGCATTTGGT CCATT

46_SE	ENSG000001977 56	*RPL37A*	ENSG00000197756,10; SE:chr2:216499398-216499832:216499866-216499949:+	0,773962	0,01399	ACGTACCAAGAAAGTCGGGA	TCCCTTTCTCCTTTGCCACA

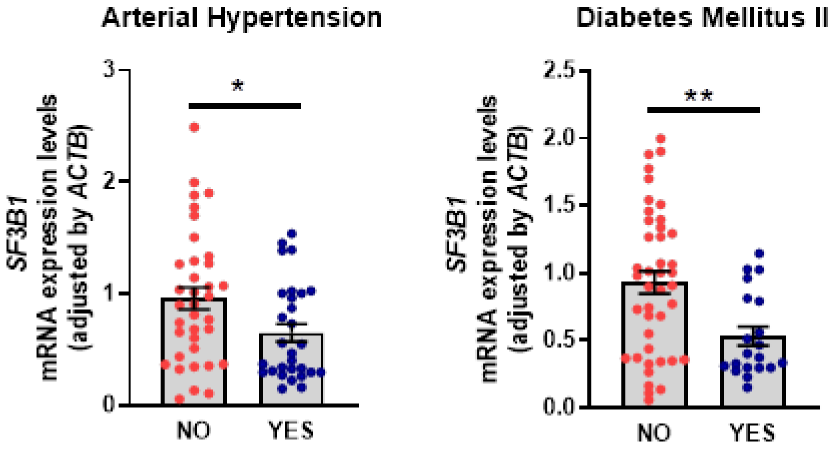

Supplemental Figure 1) *SF3B1* expression level correlation in patient without/with AHT or T2DM in PDAC FFPE samples compared with non-tumoral adjacent tissue (NTAT).

A

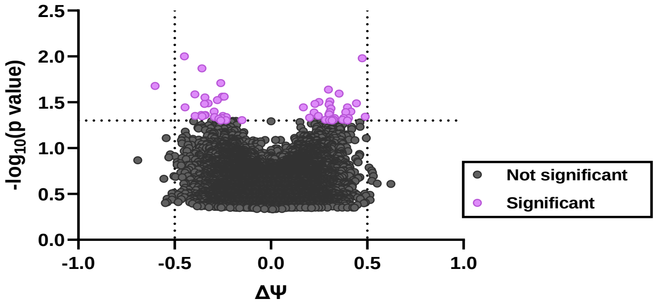

B

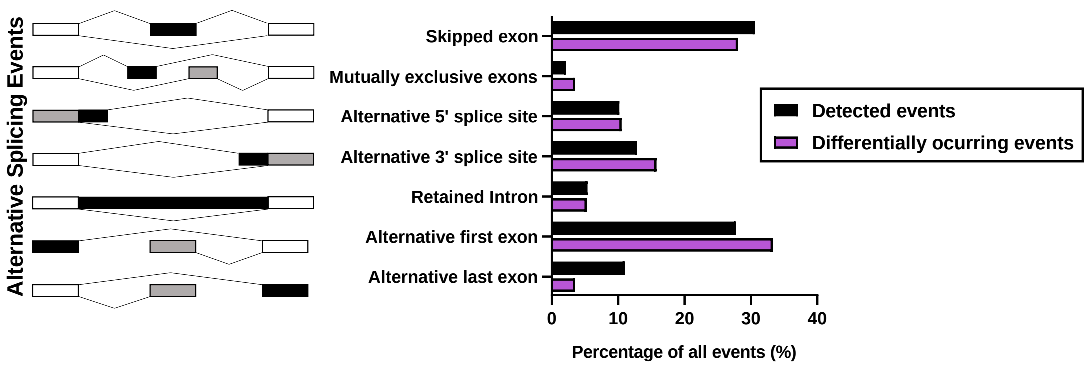

Supplemental Figure 2) A) Relationship of *SF3B1* expression levels with splicing event patterns of key genes in PDAC. **B)** Volcano-plot where ΔΨ of total events calculated is plotted against the –log10 p-value of the Fisher's Exact Test to assay differential splicing events between high and low SF3B1 expression groups of samples, showing that SF3B1 tumor expression may influence alternative splicing patterns.

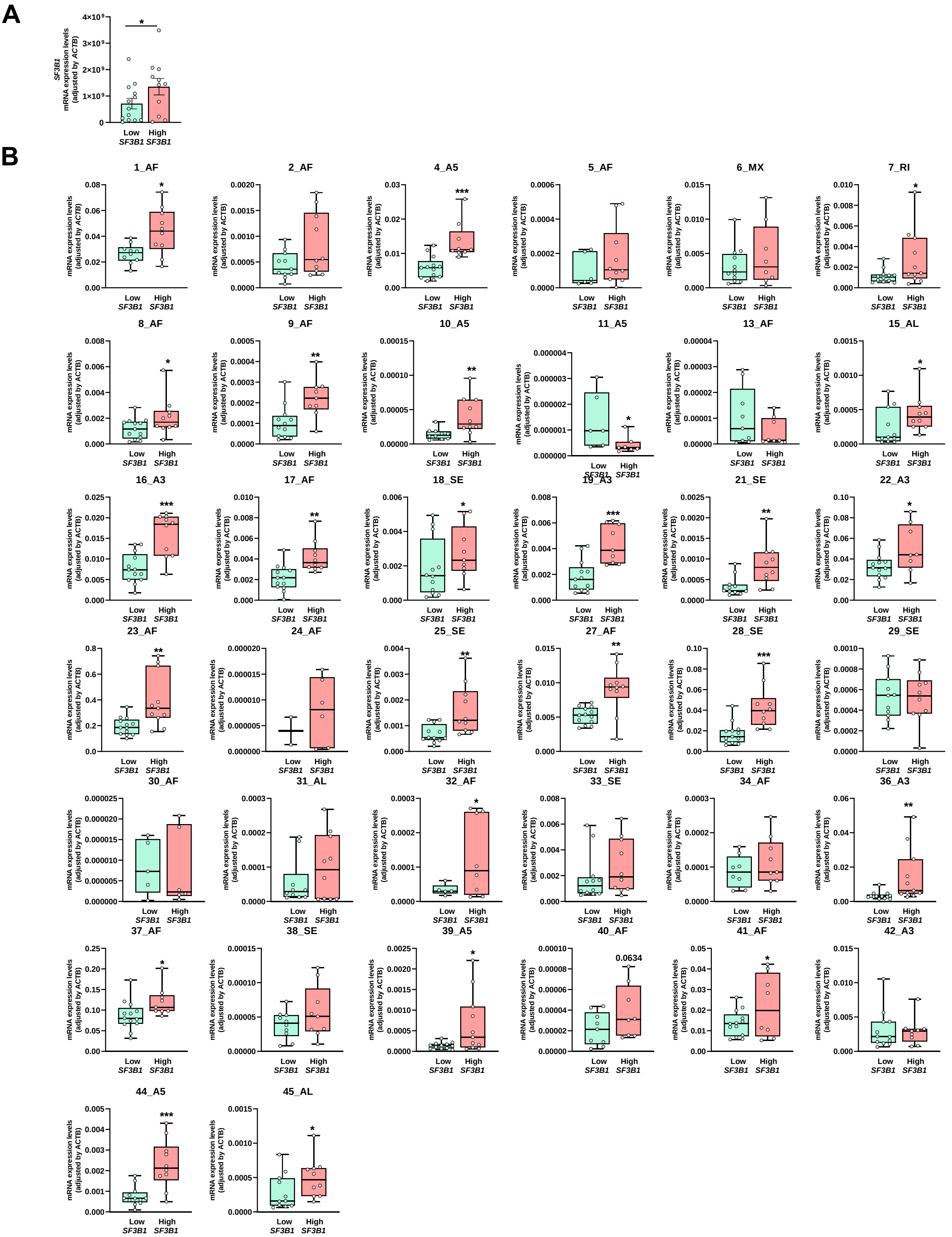

Supplemental Figure 3) A) Levels of *SF3B1* expression in PDAC samples from patients of the fresh tissue cohort in low and high *SF3B1* expression groups. **B)** Levels of expression of significantly different alternative splicing events transcripts (listed in Suppl Table 4) between High (n=11) and Low (n=13) *SF3B1* expression groups. Gene expression was normalized to *ACTB* expression. Asterisks indicate significant differences (*p < 0.05; **p < 0.01; ***p < 0.001).

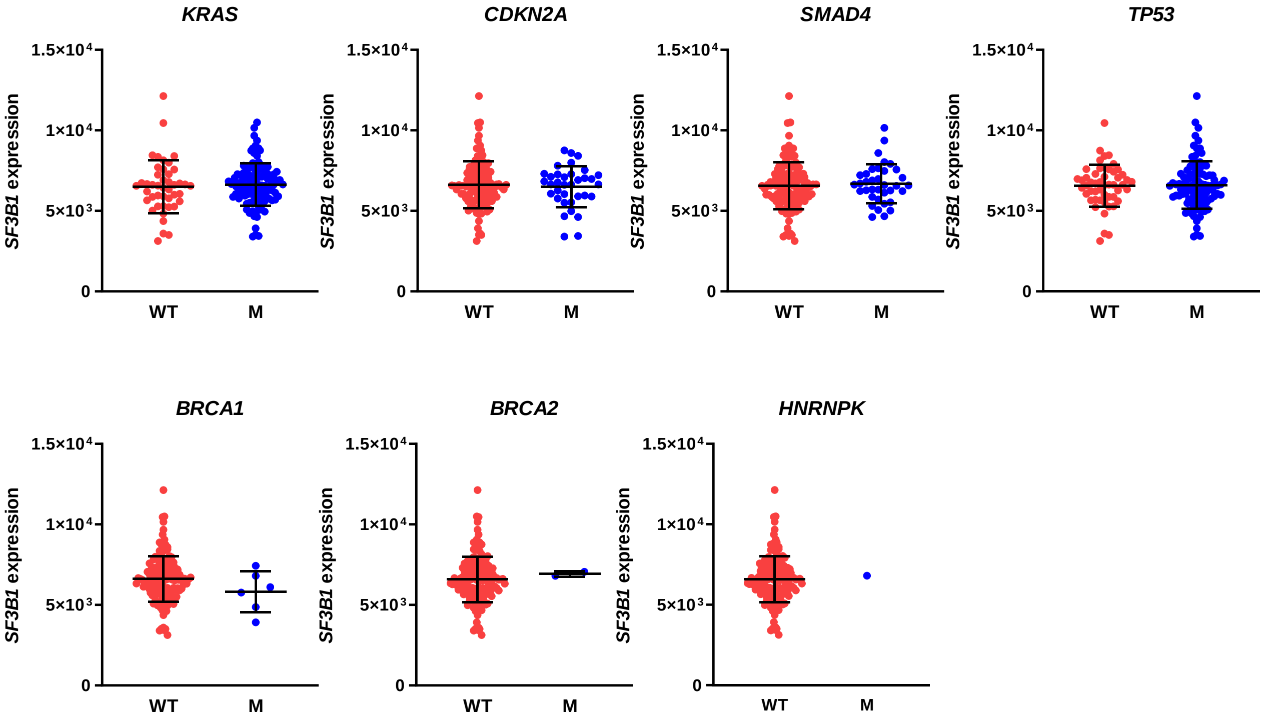

Supplemental Figure 4) Levels of *SF3B1* expression in PDAC samples from patients of the PanCancer cohort with (M) or without (WT) mutations in *KRAS, CDKN2A, SMAD4, TP53, BRCA1*, *BRCA2* and *HNRNPK*.

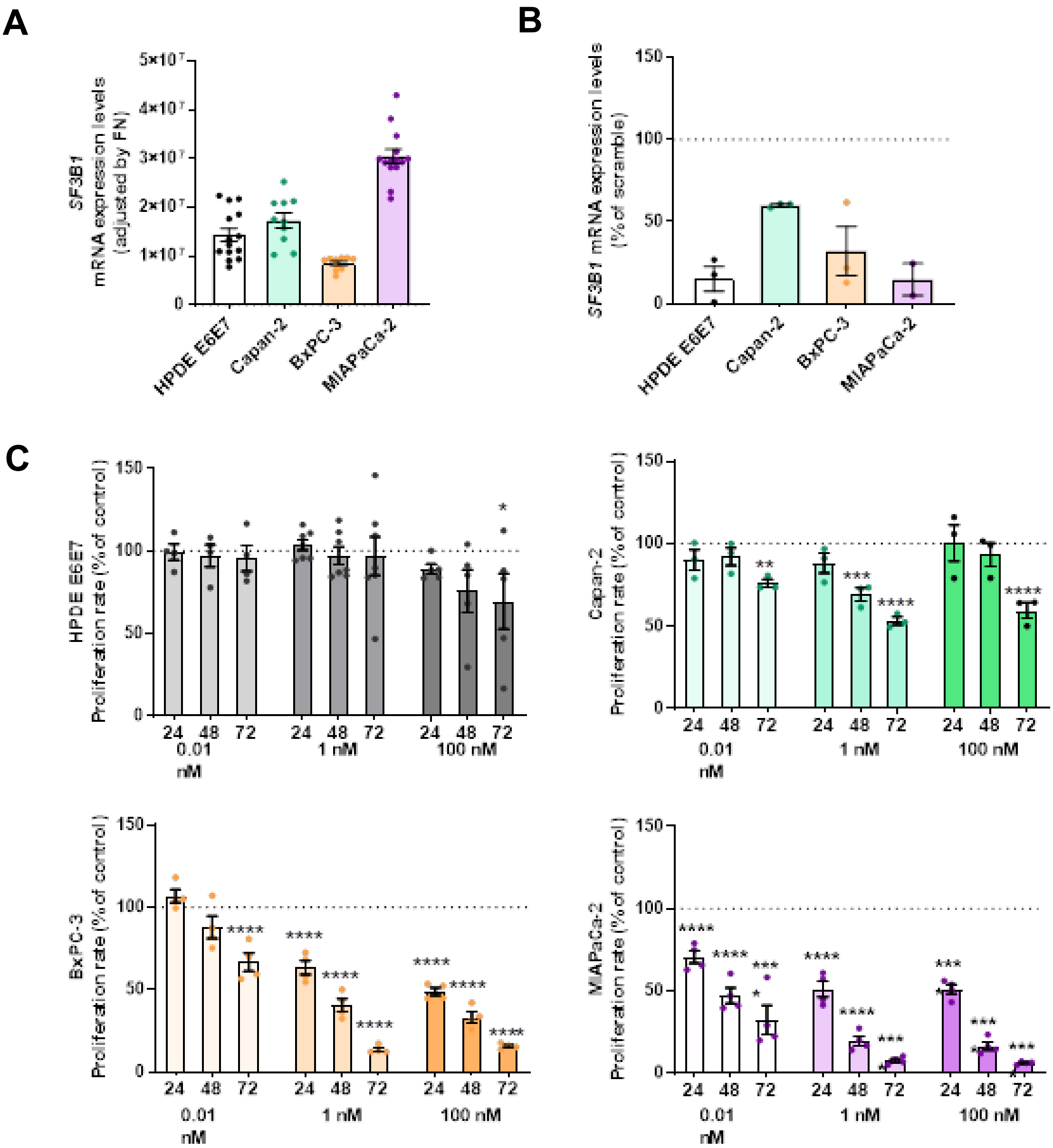

Supplemental Figure 5. Validation of SF3B1 expression silencing and optimization of Pladienolide-B dosage. A) SF3B1 basal expression levels in normal pancreatic~~s~~ HPDEE6E7 cells and Capan-2, BxPC-3 and MIAPaCa-2 PDAC cell lines adjusted by a normalization factor calculated from the expression levels of HPRT1, GAPDH and ACTB (n=5-7). **2B)** qPCR validation of the silencing efficiency achieved with SF3B1 specific siRNAs in PDAC cell lines. mRNA expression levels were normalized to ACTB expression levels. Data are expressed as a percentage of control (Scramble; set at 100%) (n=2-3). **2C)** Proliferation rates of HPDEE6E7, Capan-2, BxPC-3 and MIAPaCa-2 cell lines following treatment with different doses of Pladienolide-B compared to vehicle-treated control cells, ~~used as a control~~ (set as 100%, represented as a dotted line; n=3-5). Asterisks indicate significant differences (*p<0.05; **p<0.01; ***p<0.001).

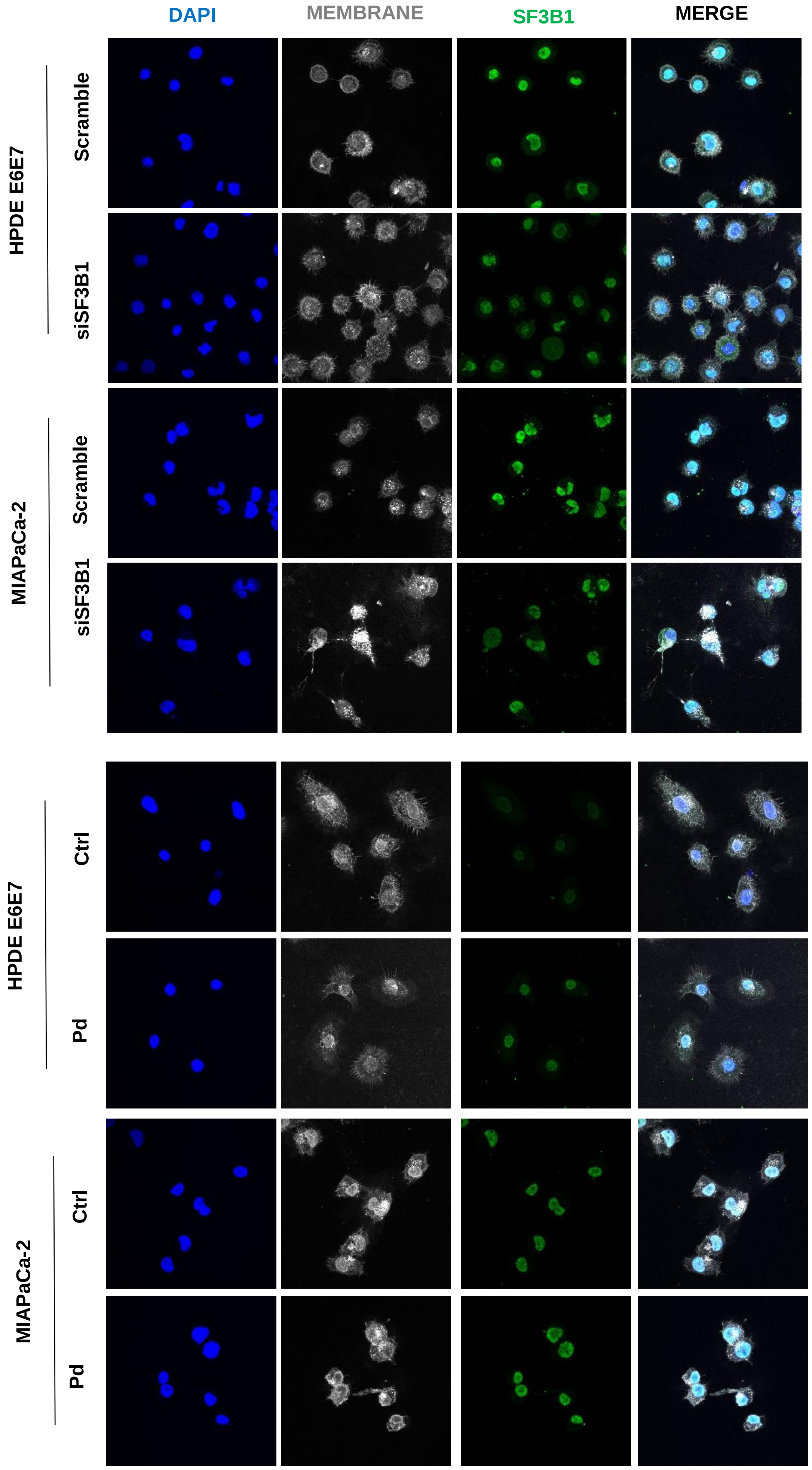
DAPI
MEMBRANE
SF3B1
MERGE
HPDE E6E7
Scramble
siSF3B1
MIAPaCa-2
Scramble
siSF3B1
HPDE E6E7
Ctrl
Pd
MIAPaCa-2
Ctrl
Pd

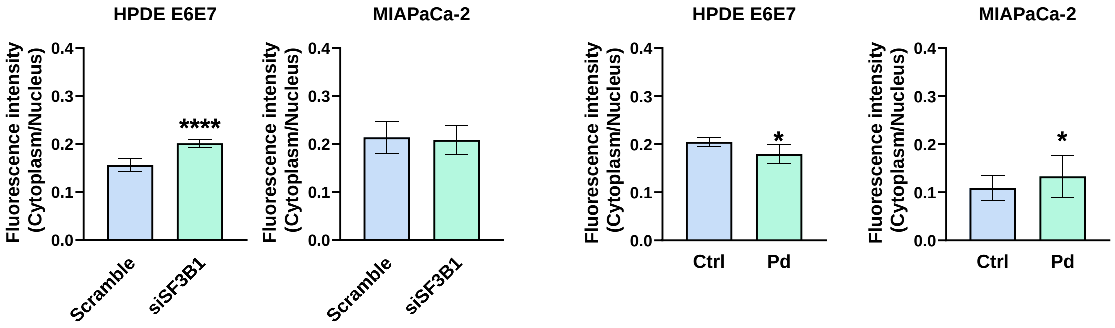

Supplemental Figure 6. SF3B1 localization in HPDE E6E7 and MIAPaCa-2 cell lines. **A)** Confocal images of Control and Pladienolide-B treated HPDE E6E7 and MIAPaCa-2 cell lines. **B)** Confocal images of Scramble and siSF3B1 in HPDE E6E7 and MIAPaCa-2 cell lines. **C)** Quantification of SF3B1 subcellular distribution in each set of samples. Asterisks indicate significant differences (*p < 0.05; **p < 0.01; ***p < 0.001).

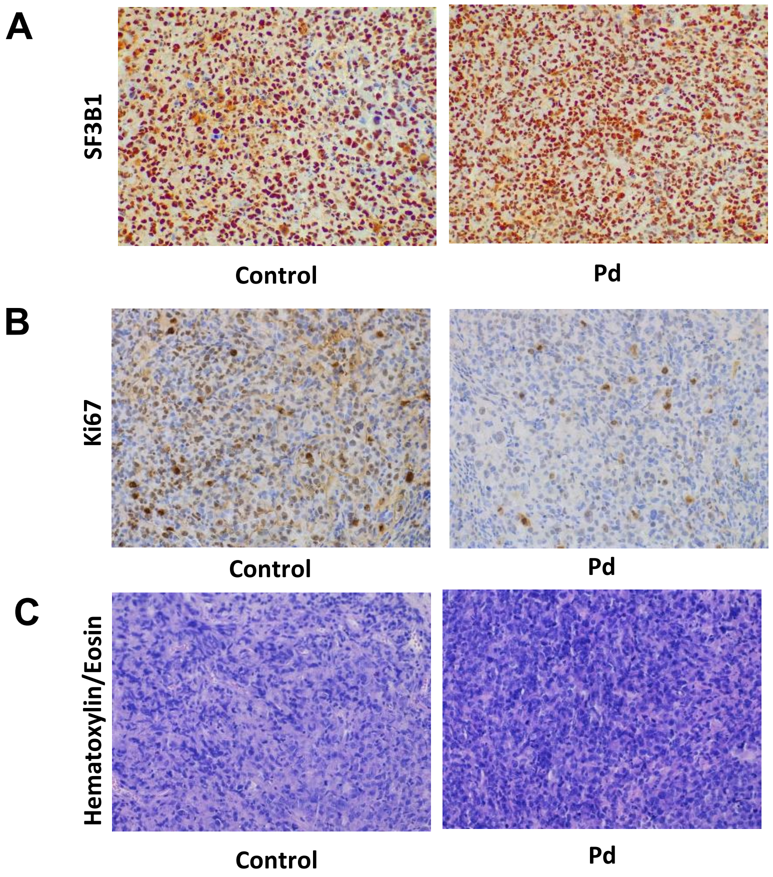

Supplemental Figure 7. Pladienolide-B effect in mice tumor xenografts. A) SF3B1 IHC analysis in vehicle (Control) and Pladienolide (Pd)-treated mice. Representative IHC 20X-images showing the general SF3B1 nuclear immunostaining in Control- and Pd-treated tumors. **B)** Ki-67 staining in representative tissue sections from tumor xenografts treated with vehicle (Control) and Pd. **C)** Hematoxylin-eosin-stained sections from vehicle (Control) and Pd-treated tumors illustrating the similarly scarce necrosis foci found in these tissues.

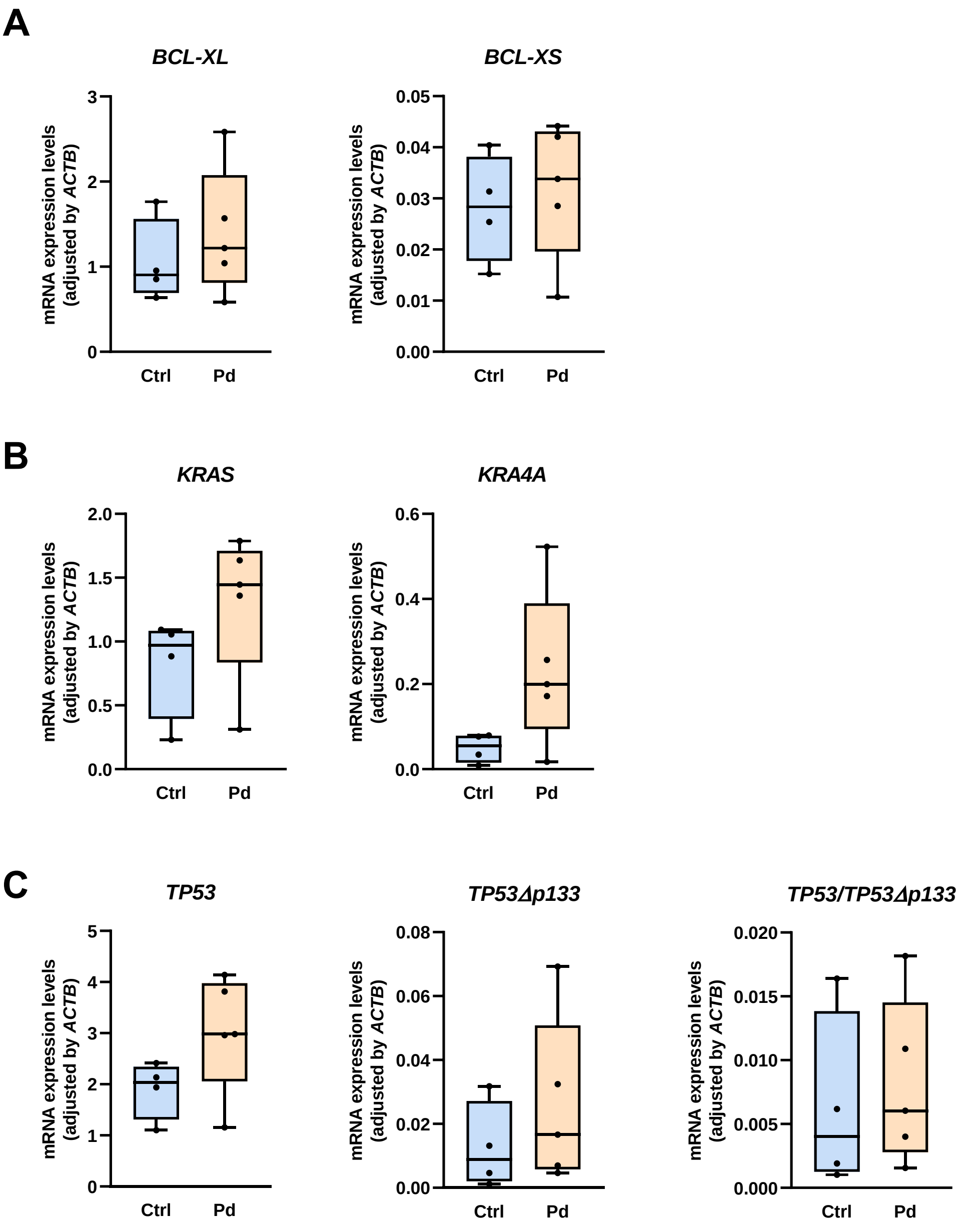

Supplemental Figure 8. Molecular profile in Pladienolide-treated mice. mRNA levels in malignancy-implicated genes. Values represent the $\log_{10}$ of expression compared with vehicle-treated (n=4). **A)** mRNA levels of *BCL-XL* and *BCL-XS* in HPDE E6E7 and MIAPaCa-2 cells treated 24h with or without (vehicle, control) Pladienolide-B (n=4). **B)** mRNA expressions of *KRAS* and *KRAS4a* in HPDE E6E7 and MIAPaCa-2 cells treated 24h with or without (vehicle, control) Pladienolide-B (n=4). **C)** Ratio of *Δ133TP53/TP53* mRNA levels in HPDE E6E7 and MIAPaCa-2 cells treated 24h with or without (vehicle, control) Pladienolide-B (n=4). Gene expression was normalized to *ACTB* expression. Asterisks indicate significant differences (*$p < 0.05$; **$p < 0.01$; ***$p < 0.001$).
